# Deletion of α7 Nicotinic Acetylcholine Receptor in POMC Neurons Impairs Energy Balance and Leptin-Induced Anorexigenic Response in Male Mice

**DOI:** 10.3390/ijms27198697

**Published:** 2026-09-29

**Authors:** Wenicios Ferreira Chaves, Suleyma de Oliveira Costa, Caroline Lobo Costa, Priscilla Karla Fernandes Lopes, Iracema Monteiro da Silva, Ísis de Cássia Alves Martins, Marciane Milanski, Letícia Martins Ignácio de Souza, Adriana Souza Torsoni, Raquel da Silva Aragão, Marcio Alberto Torsoni

**Affiliations:** 1 Laboratory of Metabolic Disorders, School of Applied Sciences, Universidade Estadual de Campinas (UNICAMP), Limeira 13484-350, Brazil; weniciosfchaves@gmail.com (W.F.C.); suleyma.costa@gmail.com (S.d.O.C.); caroline.lobo.costa@gmail.com (C.L.C.); prika_f_lopes@hotmail.com (P.K.F.L.); iramonteirosilva@gmail.com (I.M.d.S.); isismartins15@yahoo.com.br (Í.d.C.A.M.); marcia31@unicamp.br (M.M.); lmisouza@unicamp.br (L.M.I.d.S.); atorsoni@unicamp.br (A.S.T.); 2 Obesity and Comorbidities Research Center, Institute of Biology, Universidade Estadual de Campinas (UNICAMP), Campinas 13083-864, Brazil; 3 Graduate Program in Nutrition, Federal University of Pernambuco (UFPE), Recife 50670-901, Brazil; raquel.aragao@ufpe.br

**Keywords:** energy balance, glycemic homeostasis, cholinergic receptor, hypothalamus, satiety

## Abstract

Recent studies demonstrate that α7 nicotinic acetylcholine receptors (α7nAChR) in hypothalamic neurons, including pro-opiomelanocortin (POMC) neurons, modulate energy homeostasis. This study aimed to explore how the α7nAChR subunit in hypothalamic POMC neurons affects food intake and energy balance in mice. This study used site-specific knockout mice for α7nAChR in POMC neurons (Control: α7nAChR^fl/fl^; Knockout: α7nAChR^fl/fl^POMC^Cre^) generated via the Cre-LoxP system. Body weight (BW) and body weight gain (BWG) were measured. We measured energy expenditure by respirometry and assessed glycemic homeostasis using an intraperitoneal glucose tolerance test (ipGTT) and an insulin tolerance test (ipITT). Food intake was measured after intraperitoneal injection of saline (0.9%), leptin (5 μg/g BW), or the selective α7nAChR agonist PNU-282987 (1 mg/kg BW). Afterward, fasted mice were injected intraperitoneally with saline (0.9%), leptin (2.5 μg/g BW), or PNU-282987 (1 mg/kg BW), and subsequently euthanized. RT-qPCR and Western blotting analyses were performed. The α7nAChR^fl/fl^POMC^Cre^ males were initially lighter but had higher BWG. The opposite was observed in females. α7nAChR^fl/fl^POMC^Cre^ male mice showed increased epididymal fat, serum leptin levels, and food intake. Knockout male mice showed no major impairment in glucose homeostasis and signaling. Furthermore, the leptin-induced anorectic response was delayed, with reduced hypothalamic anorectic proteins and a trend toward reduced pCREB^Ser133^ after an acute leptin stimulus. PNU-282987 treatment increased pCREB^Ser133^ content and *Pcsk1* gene expression in vitro. The α7nAChR expressed in POMC neurons seems to contribute to energy and glycemic homeostasis and to the hypothalamic leptin-induced anorectic response mediated by CREB phosphorylation.

## 1. Introduction

The hypothalamus is a key region of the central nervous system (CNS) that plays a pivotal role in regulating energy homeostasis, the balance between food intake and energy expenditure [1,2]. Several hypothalamic nuclei mediate this control, including the paraventricular (PVN), lateral (LH), ventromedial (VMH), dorsomedial (DMH), and arcuate (ARC), which form an interconnected network [3,4]. Additionally, hypothalamic nuclei are strategically located around the third ventricle, a highly vascularized area that is permeable to a wide range of biomolecules [5]. The ARC is a primary center for energy metabolism, integrating peripheral feeding signals from nutrients (e.g., glucose, lipids, and protein) and hormones (e.g., leptin, insulin, cholecystokinin, and neuropeptide YY) [6]. This integration arises from a diverse neuronal population that regulates hunger and satiety signals, as well as glucose metabolism in peripheral organs, via the vagus nerve [6,7]. Several studies have identified key neuronal populations in the hypothalamic nuclei involved in metabolic regulation, including kisspeptin, Neuropeptide Y (NPY), agouti-related peptide (AgRP), and pro-opiomelanocortin (POMC) [8,9].

Energy status is reflected in circulating serum levels of certain hormones, such as insulin, leptin, and ghrelin, which can cross the blood–brain barrier and activate cells within the hypothalamus [10]. Evidence shows that hypothalamic neurons, such as POMC neurons, express insulin and leptin receptors, among others, thereby activating signaling pathways that promote negative energy balance [11,12,13]. Insulin and leptin can activate the phosphatidylinositol-3-kinase (PI3K) pathway, leading to phosphorylation of forkhead box protein O1 (FoxO1) and its export from the nucleus, allowing signal transducer and activator of transcription 3 (STAT3) to stimulate POMC expression [7,14].

Additionally, leptin can activate its canonical pathway, Janus Kinase 2 (JAK2)-STAT3, which stimulates POMC expression. In parallel, increased calcium influx into these neurons triggers phosphorylation of cAMP response element-binding protein (CREB), a transcription factor that enhances expression of genes encoding convertases (e.g., prohormone convertase and carboxypeptidase) that process POMC [15]. POMC undergoes successive cleavage into α-MSH, which binds MC4R receptors and increases sympathetic adrenergic tone in peripheral tissues [16]. Neurotransmitters like GABA, glutamate, and acetylcholine also rapidly modulate central energy homeostasis [13,17,18].

Cholinergic input in the central nervous system is widely distributed, encompassing areas that regulate physiological responses, including anxiety, central pain processing, and the anti-inflammatory response [13,19,20,21,22,23,24]. Studies have shown that a subset of nicotinic cholinergic receptors, including the alpha-7 nicotinic acetylcholine receptor (α7nAChR), is expressed in neurons and non-neuronal cells in the brain, including the hypothalamus [19,20,25]. α7nAChR is a homopentameric receptor composed of α7 subunits that serves a dual function, responding to endogenous ligands and acting as a calcium-selective ion channel [19,26]. Distinct classes of hypothalamic neurons in the ARC, such as AgRP and POMC, express α7nAChR, and their firing rates are differentially modulated by selective and non-selective agonists [13,22].

Despite these limitations, administering α7nAChR-selective agonists and/or using knockout mice has suggested direct or indirect effects on feeding behavior and glucose homeostasis [25,27,28,29]. For instance, a pharmacological approach using TC-7020, an α7nAChR agonist, has been shown to reduce body mass, food intake, and blood glucose levels in a rodent model [27]. In contrast, the constitutive absence of α7nAChR impairs various metabolic aspects, including glucose homeostasis, by compromising pancreatic development, muscle glucose uptake, and insulin sensitivity, and by increasing body fat mass and weight [28]. These results show that α7nAChR plays a key role in homeostasis, but the mechanism underlying this remains unknown.

Recently, our group demonstrated that activating α7nAChR through drugs can also trigger the hypothalamic JAK2/STAT3 pathway and reduce food intake. This regulation occurs by decreasing mRNA levels of orexigenic neuropeptides such as AgRP and NPY, while increasing expression of the anorexigenic marker POMC mRNA [25]. However, a recent study found that intraperitoneal injection of GTS-21, an α7nAChR-selective agonist, did not affect food intake or body weight in rats fed a high-fat diet (60% kcal from fat) [30]. This impairment in the anorexigenic response induced by an α7nAChR-selective agonist can be due to the hypothalamic inflammation induced by the high-fat diet, as previously demonstrated by our group [23].

The specific role of hypothalamic α7nAChR in managing energy balance and glucose stability remains uncertain. Moreover, in cases of obesity or high-fat diet (HFD) intake, α7nAChR expression may be reduced, leading to disturbances in homeostasis. So far, researchers have not studied different hypothalamic neuronal groups individually. We proposed that α7nAChR in POMC neurons could be critical for energy regulation and glucose control. To investigate this, we created a conditional knockout of the *Chrna7* gene specifically in POMC neurons via the Cre-LoxP system. We then assessed food consumption, energy expenditure, glucose metabolism, and leptin signaling in these mice fed a standard chow diet.

## 2. Results

### 2.1. Deletion of α7nAChR in Hypothalamic POMC Neurons Leads to Molecular Changes Linked to Energy Balance Regulation in Peripheral Tissues and to Hypothalamic Neuropeptide Expression, While Increasing Food Intake During the Dark Cycle in Male Mice

**Figure 1:**
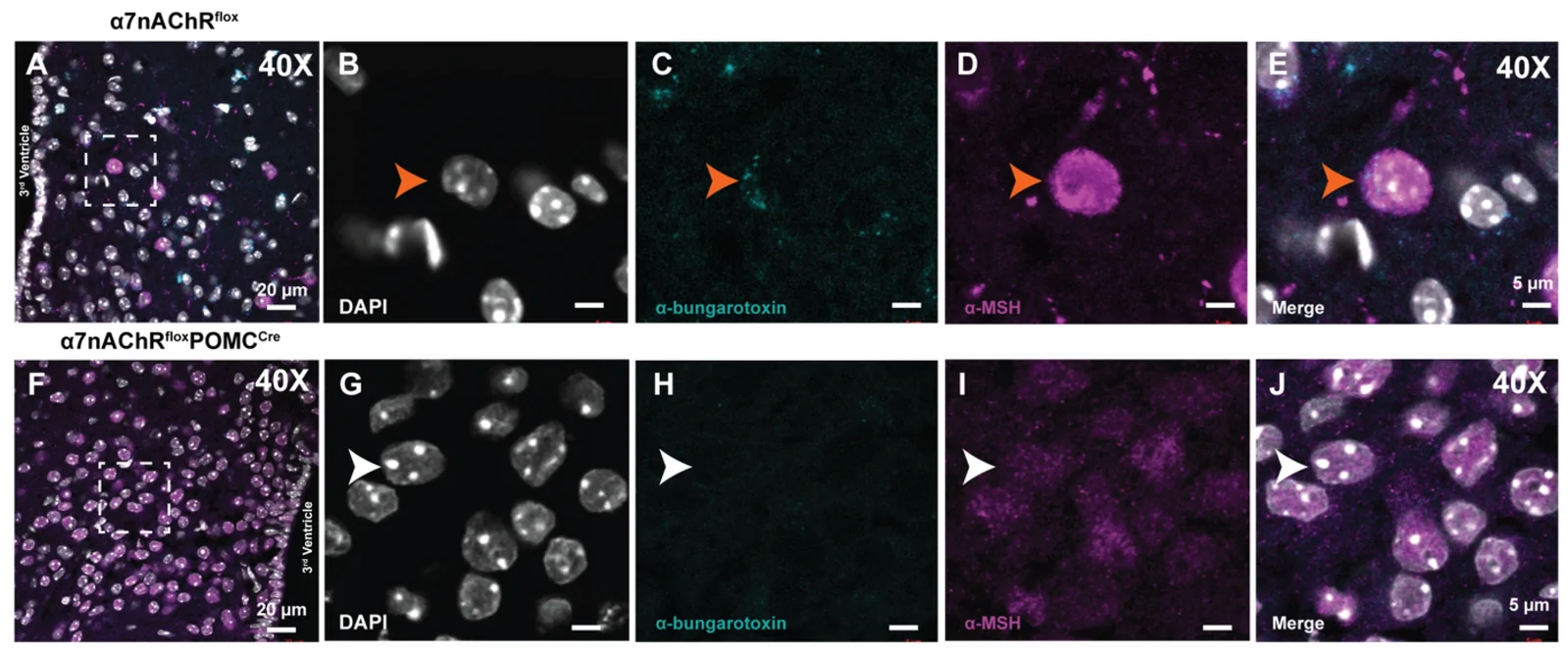
Deletion of α7nAChR in POMC hypothalamic neurons was confirmed through immunofluorescence. Coronal hypothalamic mouse brain slices (15 μm) were incubated with specific antibodies conjugated with a fluorochrome, and the nucleus was labeled with DAPI. Images were taken at lower magnification from both groups (**A**,**F**). Zoom images were taken from an α7nAChR^flox^ mouse (control) to show POMC neurons co-localizing with α7nAChR (orange arrow; (**B**–**E**)) or an α7nAChR^flox^POMC^Cre^ mouse (knockout) showing no co-localization with α7nAChR (white arrow; (**G**–**J**)). Dashed square marks the zoomed area.

The deletion of α7nAChR was confirmed by immunofluorescence with an anti-α-MSH antibody to identify POMC neurons and α-bungarotoxin, a specific α7nAChR ligand. As shown in Figure 1, POMC neurons (α-MSH^+^ cells) expressing α7nAChR (α-bungarotoxin^+^ cells) were detected in α7nAChR^flox^ mice (CT). However, in α7nAChR^fl/fl^POMC^cre^ mice, the α-MSH^+^ cells did not show immunofluorescence for α-bungarotoxin (Figure 1).

**Figure 2:**
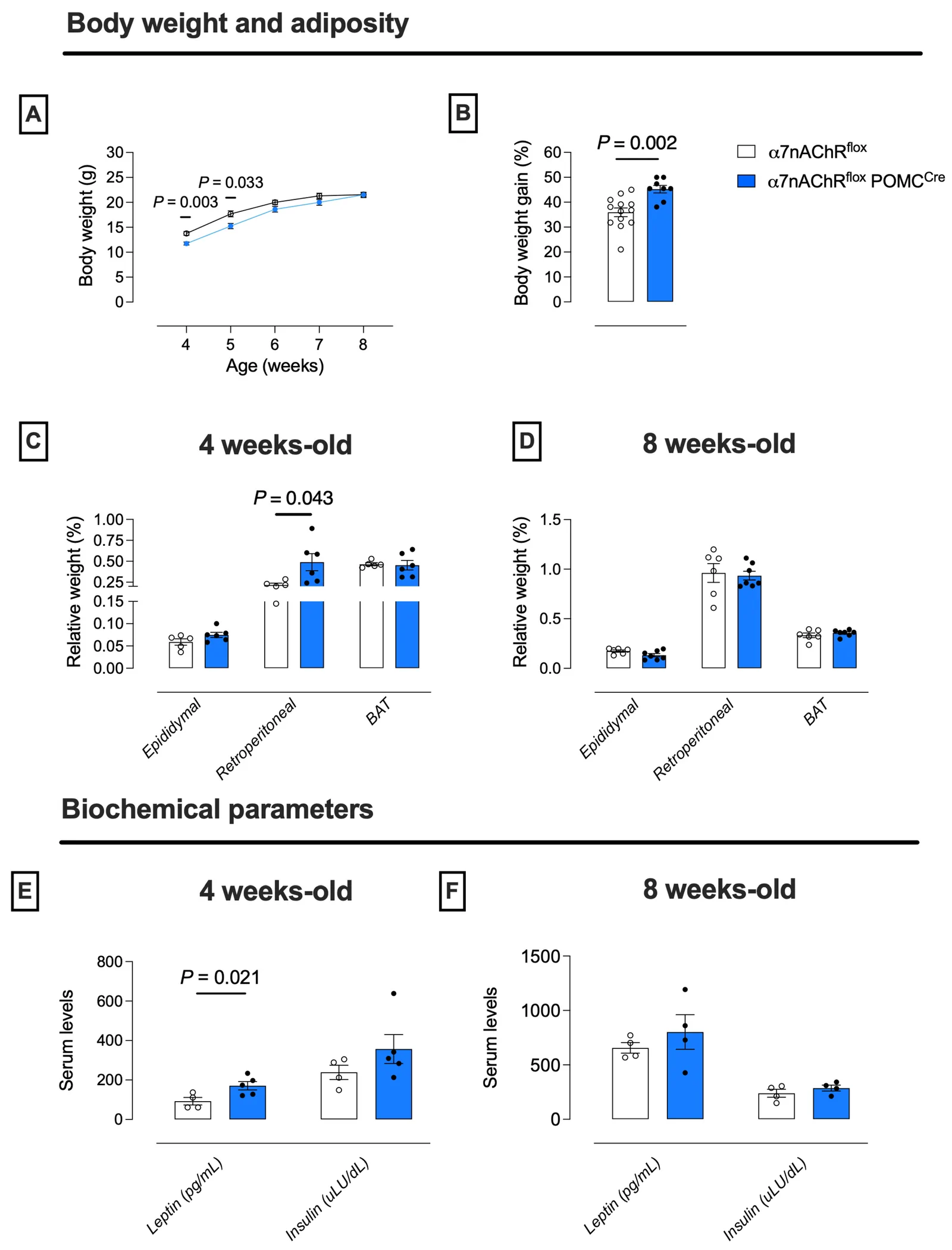
Growth parameters, adiposity, leptin, and insulin serum levels in males at the 4th and 8th week of life. Body weight (**A**) was measured weekly from the 4th until the 8th week of life, while body weight gain (**B**) was measured at the 8th week of life (*n* = 8–13). Adiposity was determined by weighing the epididymal, retroperitoneal, and brown adipose tissue (**C**,**D**) (*n* = 5–7). We measured serum leptin and insulin levels by ELISA kits at the same ages (**E**,**F**) (*n* = 4–5). Data were expressed as mean ± S.E.M.; Welch’s *t*-test was performed, except for the growth curve, for which Two-way ANOVA followed by Bonferroni’s post hoc was performed. Filled and empty circles represent individual plots.

The dynamics of body weight (BW) and gain (BWG), white adipose tissue mass, and serum leptin and insulin were evaluated in males (Figure 2) and females (Supplementary Figure S1) from the 4th to the 8th week of life. α7nAChR^flox^POMC^cre^ male mice were lighter than controls at the 4th and 5th weeks but had a higher overall BWG (Figure 2A,B). α7nAChR^flox^POMC^cre^ male mice had a heavier retroperitoneal fat pad at the 4th week and higher serum leptin levels (Figure 2C,E). In contrast, α7nAChR^flox^POMC^cre^ female mice were heavier than controls at the 4th and 5th weeks but had lower BWG overall (Supplementary Figure S2A,B).

**Figure 3:**
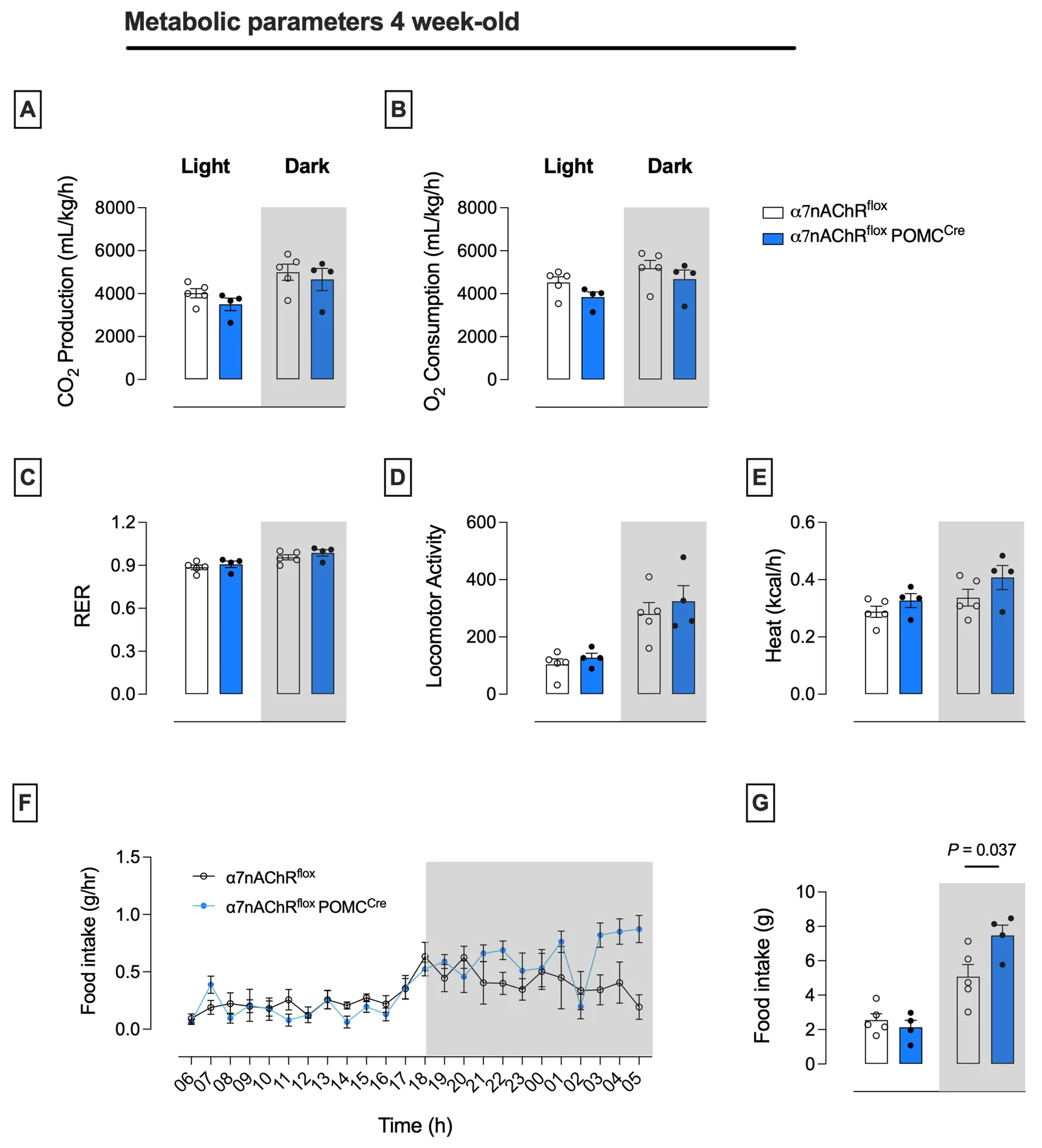
Metabolic parameters in male mice at the 4th week of life. Metabolic parameters, including oxygen consumption (**A**), carbon dioxide production (**B**), RER (**C**), locomotor activity (**D**), heat (**E**), and food intake during 24 h (**F**) and per cycle (**G**), were measured by indirect calorimetry for 24 h (*n* = 4–5). Data were expressed as mean ± S.E.M.; Welch’s *t*-test was performed, except for the food intake curve, for which Two-way ANOVA followed by Bonferroni’s post hoc was performed. Filled and empty circles represent individual plots.

**Figure 4:**
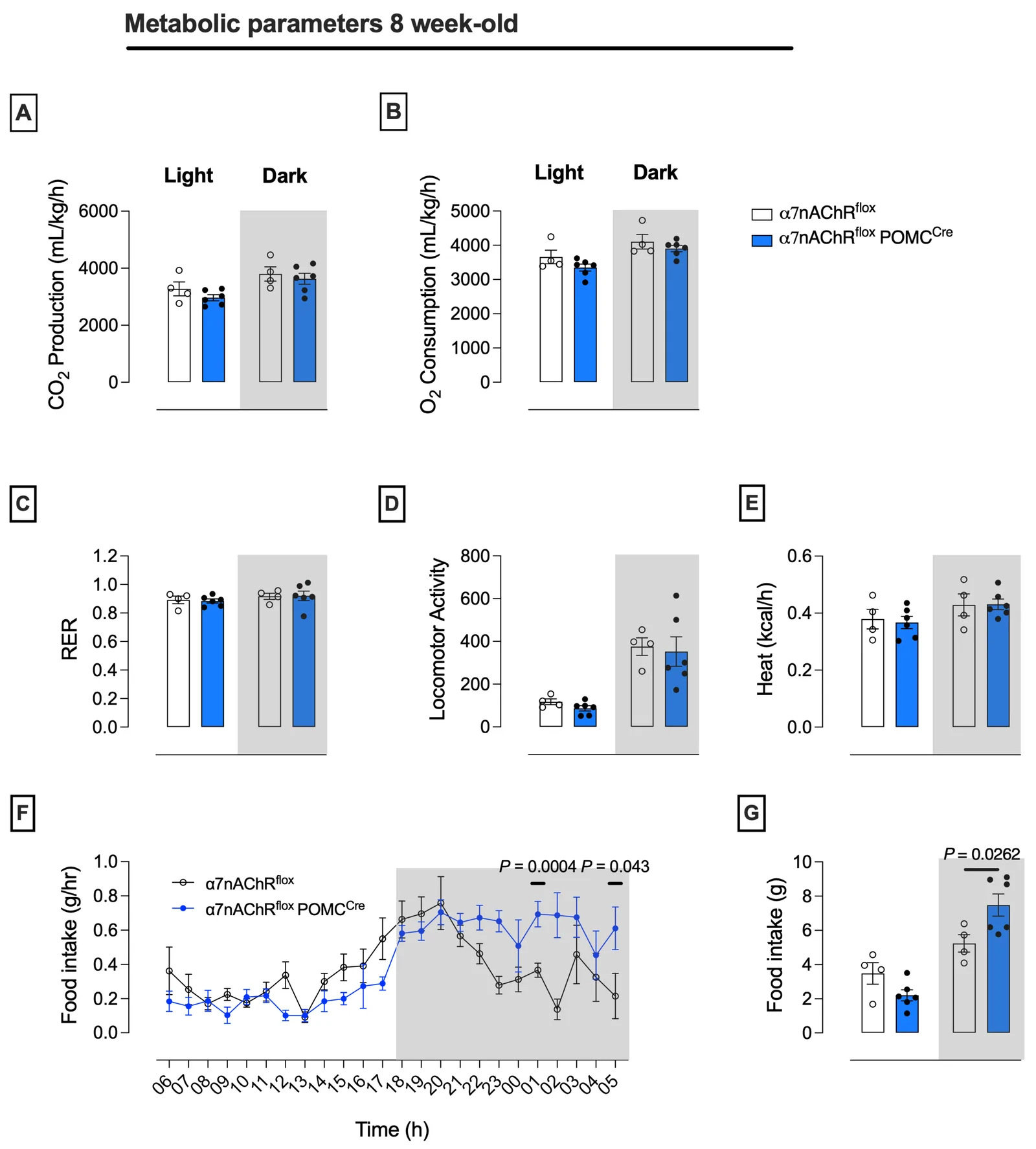
Metabolic parameters in male mice at the 8th week of life. Metabolic parameters, including oxygen consumption (**A**), carbon dioxide production (**B**), RER (**C**), locomotor activity (**D**), heat (**E**), and food intake during 24 h (**F**) and per cycle (**G**), were measured by indirect calorimetry for 24 h (*n* = 4–5). Data were expressed as mean ± S.E.M.; Welch’s *t*-test was performed, except for the food intake curve, for which Two-way ANOVA followed by Bonferroni’s post hoc was performed. Filled and empty circles represent individual plots.

Given the crucial role of POMC neurons in energy homeostasis and food intake, we assessed metabolic parameters by indirect calorimetry at the 4th and 8th weeks of life in males (Figure 3 and Figure 4) and females (Supplementary Figures S2 and S3). Deletion of α7nAChR specifically in POMC neurons did not significantly affect O_2_ consumption, CO_2_ production, respiratory exchange ratio (RER), locomotor activity, or heat production in male mice at both ages (Figure 3 and Figure 4). However, given that we do not have data on lean and fat mass at the time of testing and that calorimetry was performed for only 24 h, these data do not allow us to conclude that the genotypes do not differ in the assessed parameters. α7nAChR^flox^POMC^cre^ female mice showed lower locomotor activity during the light cycle than α7nAChR^flox^ mice at the 8th week (Supplementary Figure S2D). α7nAChR^flox^POMC^cre^ male mice showed a higher food intake during the dark cycle at the 4th and 8th weeks (Figure 3G).

Because we observed changes in adiposity and leptin levels in male mice but not in females, we focused our analysis on molecular disruptions related to energy homeostasis in males. Accordingly, we collected peripheral tissues, including white adipose tissue (WAT) and brown adipose tissue (BAT), to investigate potential molecular impairments underlying the observed phenotype.

**Figure 5:**
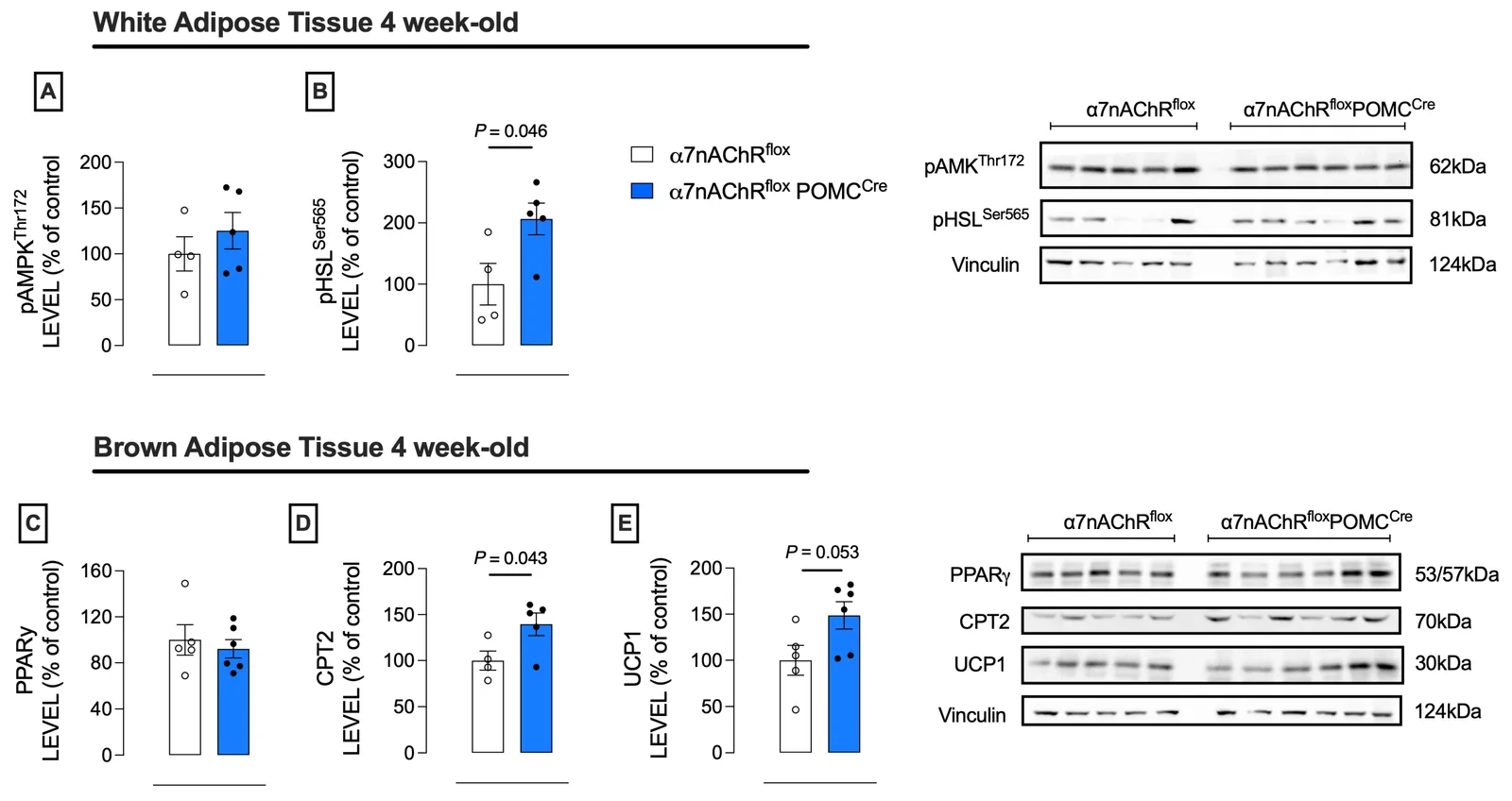
Metabolic markers in brown and white adipose tissues in male mice at the 4th week of life. Epididymal white adipose tissue from male mice was collected to evaluate metabolic markers, including pAMPK^Thr172^ (**A**) and pHSL^Ser565^ (**B**) (*n* = 4–5). The protein content of PPARγ (**C**), carnitine palmitoyltransferase 2 (**D**), and uncoupling protein 1 (**E**) were measured in the brown adipose tissue (*n* = 4–5). Protein content was normalized using an antibody against vinculin. Data were expressed as mean ± S.E.M., Welch’s *t*-test was performed. Samples were excluded when they could not be quantified (*n* = 1 from each group). Filled and empty circles represent individual plots.

The epididymal white adipose tissue (eWAT) from knockout mice exhibited increased protein levels of pHSL^Ser565^, an inhibitory regulatory site of this enzyme (Figure 5B). Furthermore, α7nAChR^flox^POMC^cre^ mice showed high protein levels of Carnitine Palmitoyltransferase II (CPT2) and a trend toward increased Uncoupling protein 1 (UCP1) in the BAT at the 4th week (Figure 5D,E).

**Figure 6:**
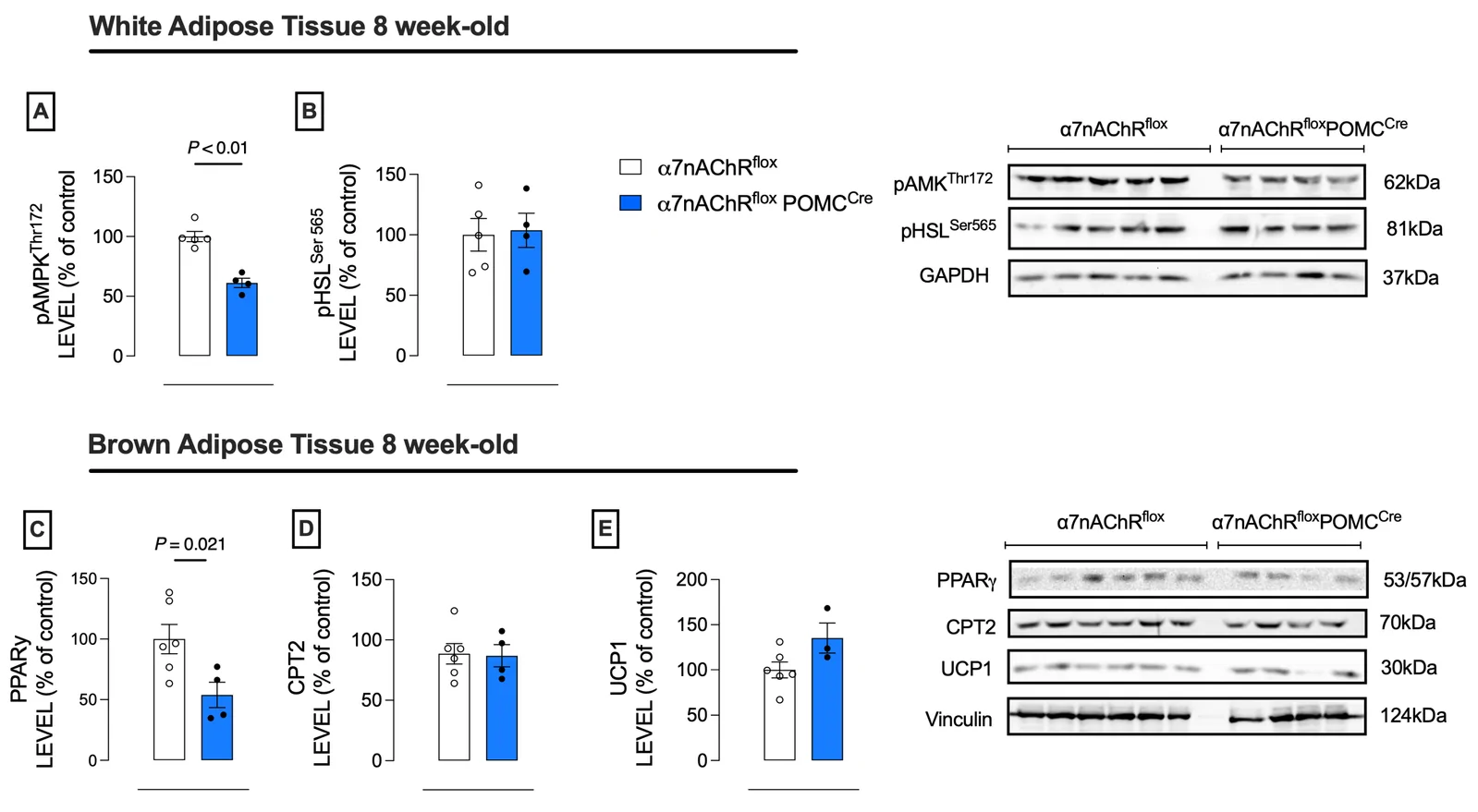
Metabolic markers in the brown and white adipose tissues in male mice at the 8th week of life. Epididymal white adipose tissue from male mice was collected to assess metabolic markers, including pAMPK^Thr172^ (**A**) and pHSL^Ser565^ (**B**) (*n* = 4–5). The protein content of PPARγ (**C**), carnitine palmitoyl transferase 2 (**D**), and uncoupling protein 1 (**E**) were measured in the brown adipose tissue (*n* = 4–6). Protein content was normalized using antibodies against GAPDH or vinculin. Data were expressed as mean ± S.E.M., Welch’s *t*-test was performed. Samples were excluded when they could not be quantified (*n* = 1 from α7nAChRfloxPOMCcre). Filled and empty circles represent individual plots.

The α7nAChR^flox^POMC^cre^ mice showed a reduction in the protein levels of pAMPK^Thr172^ in the eWAT at the 8th week (Figure 6A). Additionally, PPARγ protein levels were decreased in the BAT (Figure 6C). These findings suggest molecular alterations related to energy balance maintenance in peripheral tissues of male mice with constitutive deletion of α7nAChR in POMC neurons.

**Figure 7:**
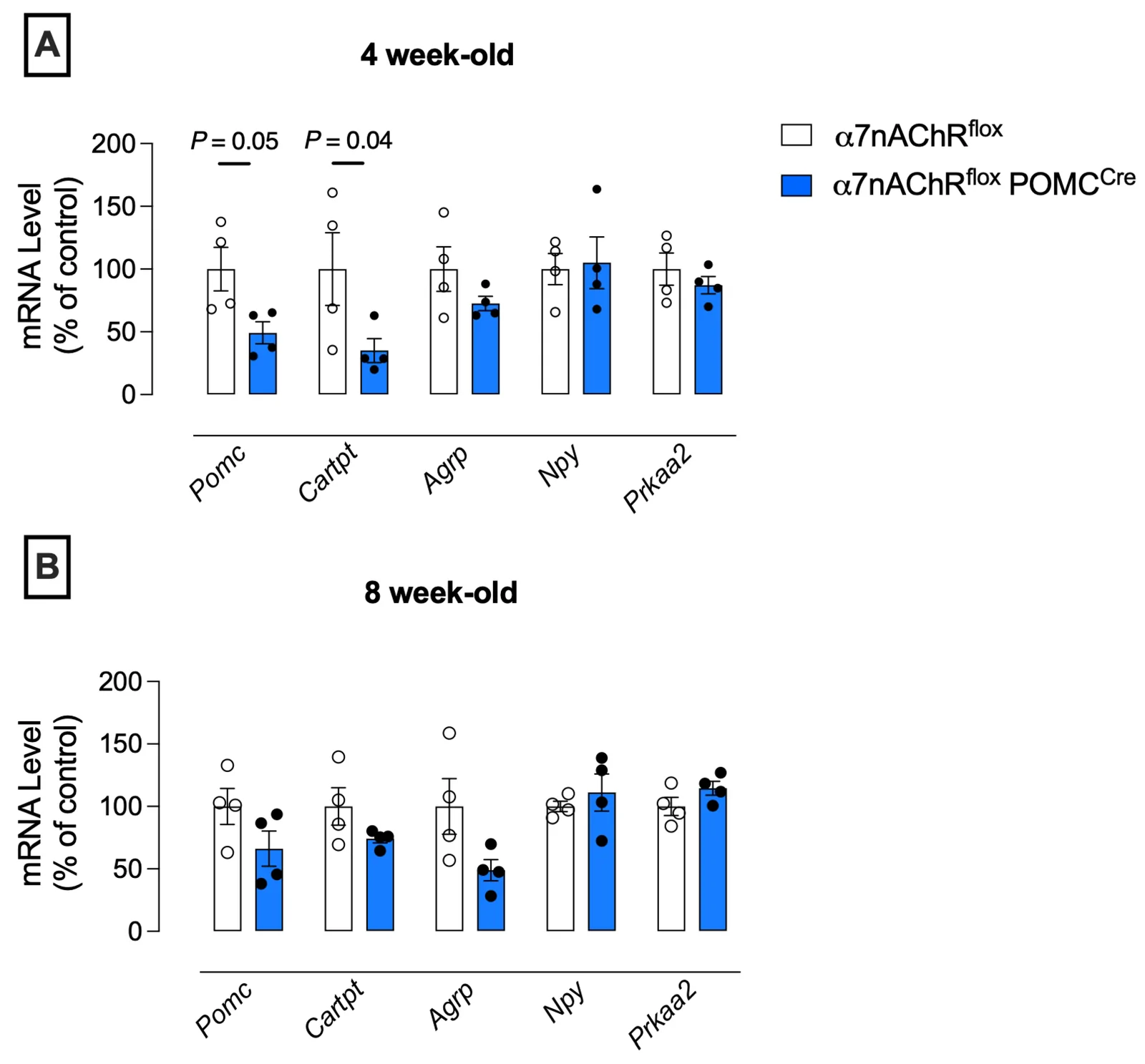
Hypothalamic neuropeptide mRNA expression in α7nAChR^flox^POMC^Cre^ and α7nAChR^flox^ male mice. mRNA expression levels of anorexigenic and orexigenic neuropeptides in the hypothalamus were measured in male mice fed a chow diet on the 4th (**A**) and 8th weeks of life (**B**) (*n* = 4). mRNA levels were normalized using *Actb*. Reactions were performed in triplicate. Data are expressed as the mean ± S.E.M. Welch’s *t*-test was performed. Filled and empty circles represent individual plots.

Gene expression of neuropeptides involved in regulating food intake in the hypothalamus of male mice was analyzed at the 4th and 8th weeks of life. Our findings showed that *Cartpt* was less expressed in the hypothalamus of α7nAChR^flox^POMC^cre^ male mice than in α7nAChR^flox^ mice at the 4th week of life, and the *Pomc* gene showed a trend (Figure 7A). However, the expression of other genes evaluated, such as *Npy*, *Agrp*, and *Prkaa2*, remained unaffected.

### 2.2. α7nAChR Expressed in POMC Hypothalamic Neurons Appears Not to Be Relevant to Hepatic Glucose Homeostasis in Male Mice

**Figure 8:**
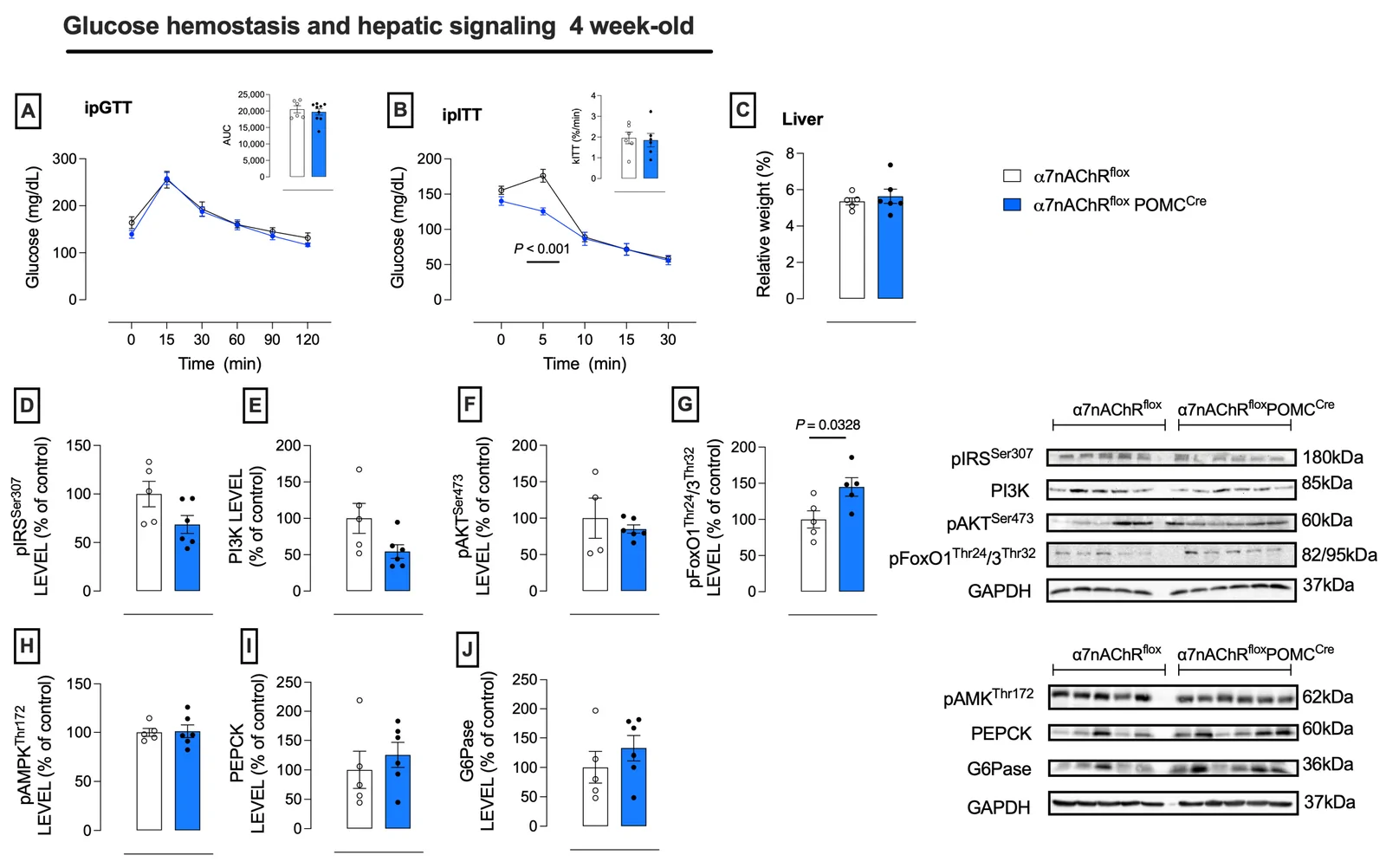
Hepatic glucose homeostasis parameters in male mice at 4th weeks of age. Glucose homeostasis was evaluated by ipGTT (**A**) and ipITT (**B**) after weaning (*n* = 6–9), and the relative liver weight was measured (**C**) (*n* = 5–6). Hepatic protein content of enzymes involved in insulin signaling (**D**–**H**) and gluconeogenesis was measured (**I**,**J**) (*n* = 5–6). Protein content was normalized to GAPDH. Data are expressed as mean ± S.E.M. Welch’s *t*-test was performed, except for ipGTT and ipITT, for which Two-way ANOVA followed by Bonferroni’s post hoc was performed. We excluded samples that could not be quantified (*n* = 1 from α7nAChR^flox^POMC^cre^). Filled and empty circles represent individual plots.

**Figure 9:**
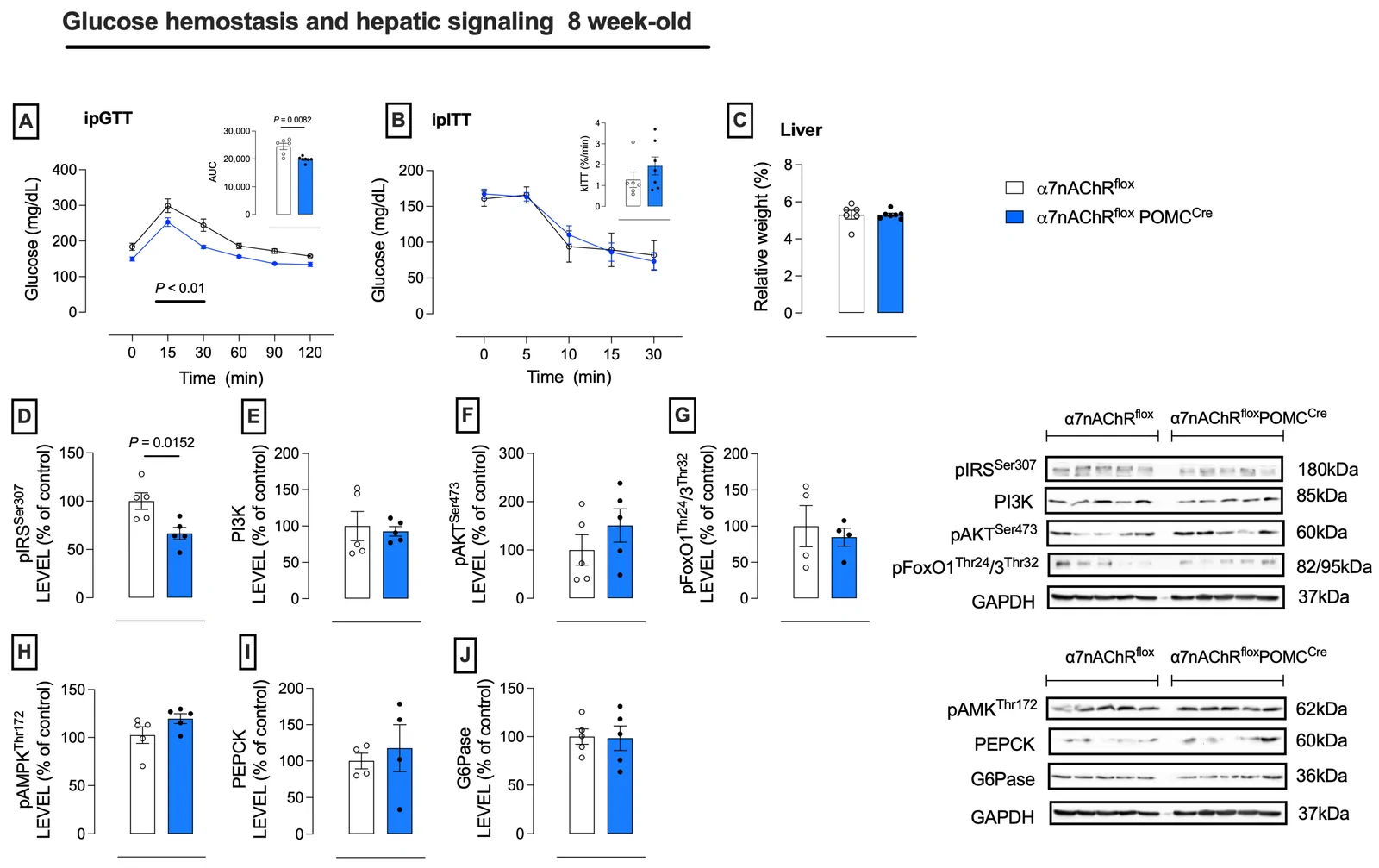
Hepatic glucose homeostasis parameters in male mice at the 8th week of age. Glucose homeostasis was evaluated by ipGTT (**A**) and ipITT (**B**) after weaning (n = 6–7), and the relative liver weight was measured (**C**) (*n* = 6–7). Hepatic protein content of enzymes involved in insulin signaling (**D**–**H**) and gluconeogenesis was measured (**I**,**J**) (*n* = 5). Protein content was normalized to GAPDH. Data are expressed as mean ± S.E.M. Welch’s *t*-test was performed, except for ipGTT and ipITT, for which Two-way ANOVA followed by Bonferroni’s post hoc was performed. We excluded samples that could not be quantified (*n* = 1 from each group). Filled and empty circles represent individual plots.

POMC hypothalamic neurons play a relevant role in hepatic glucose homeostasis. We then investigated whether deleting α7nAChR in these neurons could impair hepatic glucose homeostasis at 4 and 8 weeks of life. The ipGTT assessment remained unaffected. However, knockout mice, compared to controls, showed an ipITT curve indicating a rapid insulin effect in decreasing glucose at 5 min in the 4th week (Figure 8B). Additionally, these mice presented a higher hepatic pFoxO1/3^Thr24/32^ protein content (Figure 8G). We re-evaluated these parameters at the 8th week of life. The glucose levels and AUC in the ipGTT were lower in α7nAChR^flox^ POMC^Cre^ mice (Figure 9A), as was the protein content of pIRS^Ser307^ (Figure 8D). Together, these data indicate that α7nAChR deletion in POMC neurons does not cause major impairments in glucose homeostasis.

### 2.3. Deletion of α7nAChR in Hypothalamic POMC Neurons Is Associated with a Delayed Anorexigenic Response to Leptin, Potentially Driven by Reduced pCREB^Ser133^ Levels, but Not by Changes in pSTAT3^Tyr705^ or acSTAT3^Lys685^

**Figure 10:**
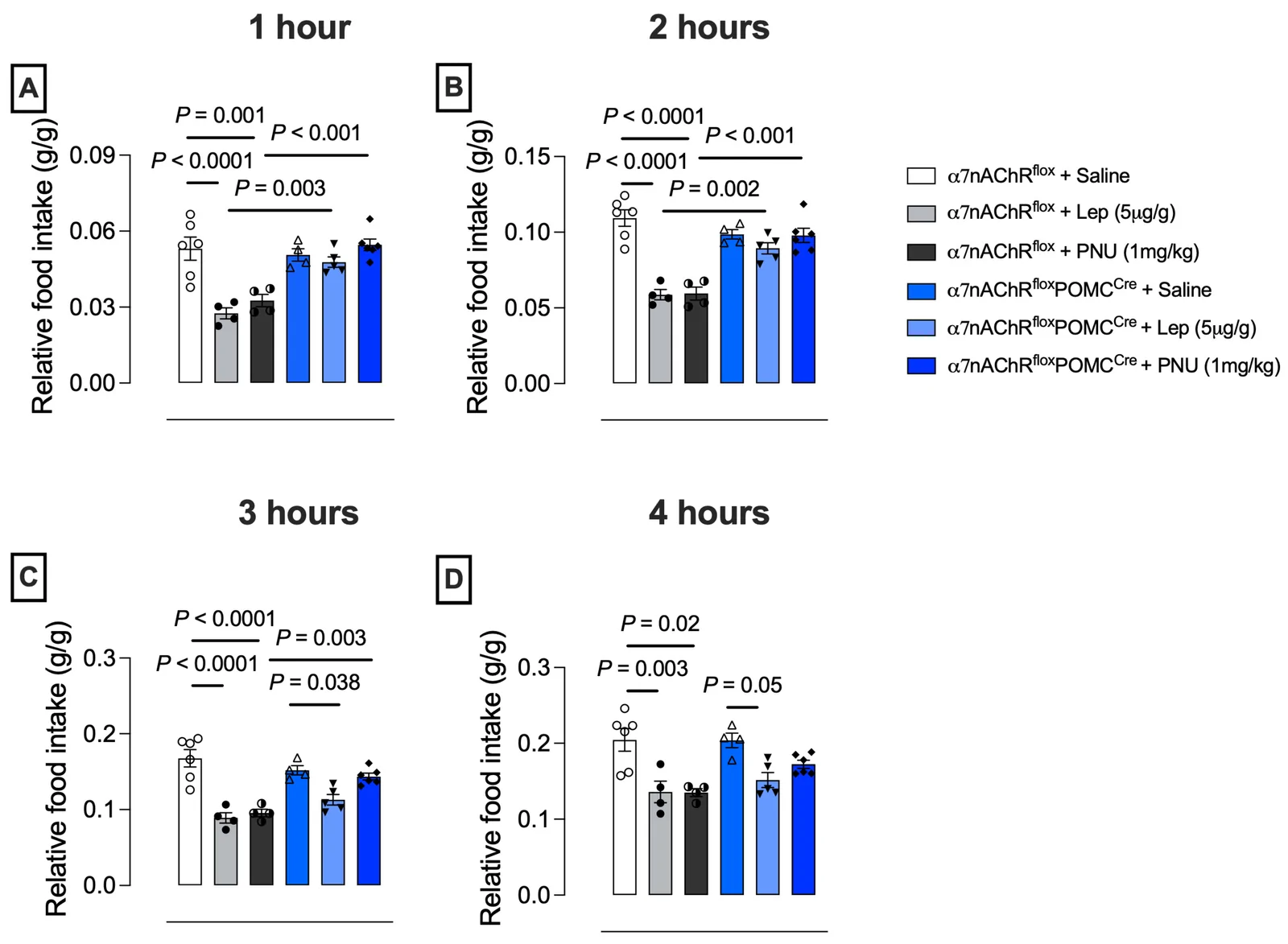
Relative food intake from animals stimulated with leptin or PNU-282987 by intraperitoneal injection. After fasting for 24 h, male mice received a single dose of filtered saline solution (0.9%) or leptin (5 μg/g BW), or PNU-282987 (1 mg/kg BW) by intraperitoneal injection. Then, we measured relative food intake at 1, 2, 3, 4, and 24 h after the stimulus (**A**–**D**) (*n* = 4–6). Data are expressed as the mean ± S.E.M. Two-way ANOVA followed by Bonferroni’s post hoc was performed. Interaction: drug × genotype at 1 h (*p* = 0.009), 2 h (*p* < 0.0001), 3 h (*p* = 0.017), and 4 h (*p* = 0.2403). Filled and empty circles and triangles represent individual plots.

Next, we evaluated whether deleting α7nAChR in POMC neurons could impair food intake in male mice. Male mice were fasted for 24 h, then, 30 min before the dark cycle, received a single intraperitoneal dose of filtered saline (0.9% *w*/*v*), leptin (5 μg/g BW), or PNU-282987 (1 mg/kg BW). We measured relative food intake at 1, 2, 3, and 4 h after the stimuli. In α7nAChR^flox^ mice, leptin or PNU-282987 injection reduced food intake from 1 to 4 h after injection (Figure 10A–D). In α7nAChR^flox^POMC^cre^ mice, leptin reduced food intake only 3 h after the leptin stimulus (Figure 10C,D). Acute intraperitoneal injection of PNU-282987 had no effect in α7nAChR^flox^POMC^cre^ mice. The drug × genotype × interaction at 1 h (*p* = 0.009), 2 h (*p* < 0.0001), 3 h (*p* = 0.017), and 4 h (*p* = 0.2403) suggests a delayed anorexigenic response.

**Figure 11:**
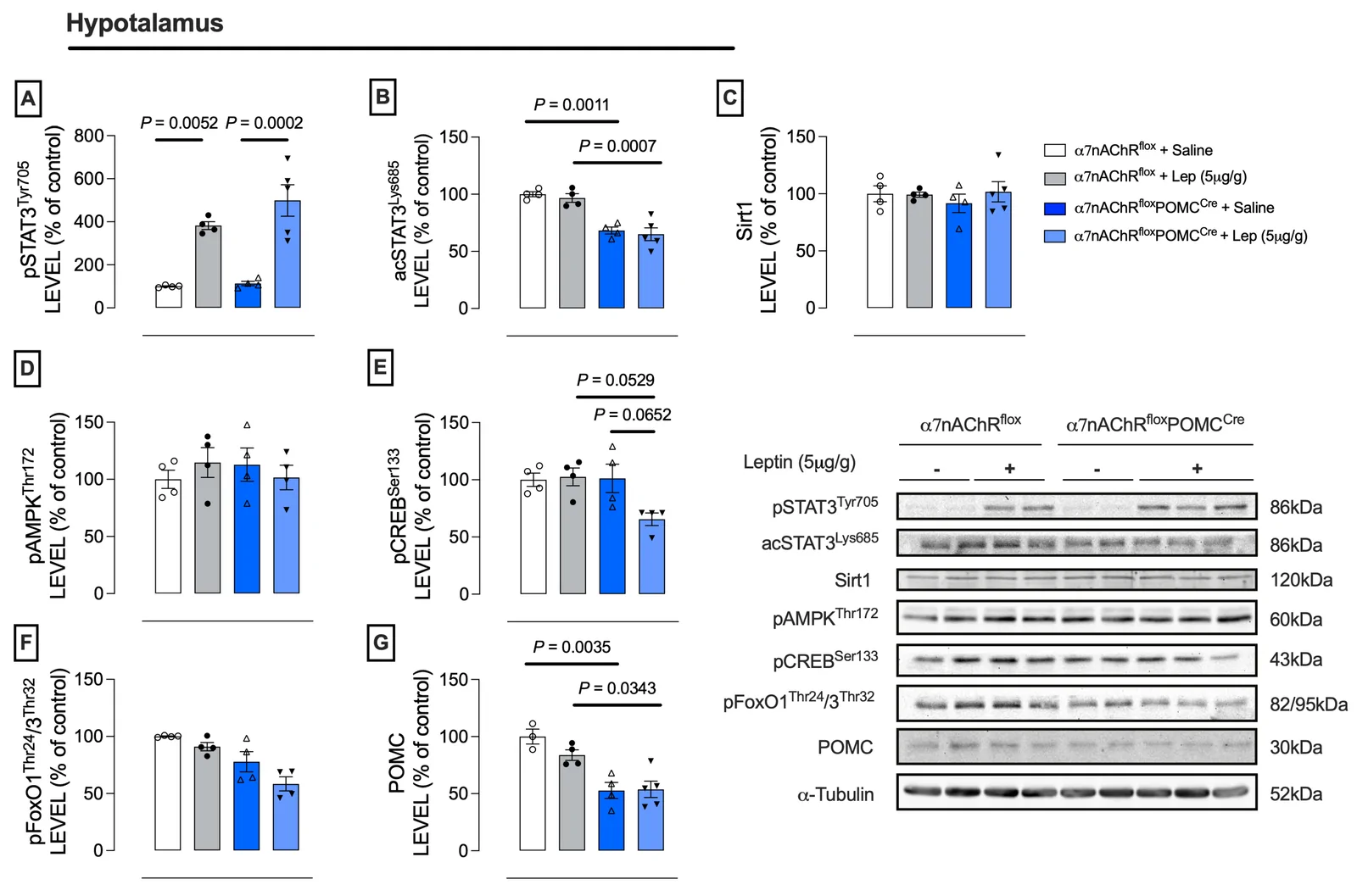
Hypothalamic proteins involved in the anorexigenic response to leptin administered by intraperitoneal injection. Mice were fasted overnight and then received a single intraperitoneal injection of saline solution (0.9%) or leptin (2.5 μg/g BW) (*n* = 4–5). The hypothalamus was collected 30 min after the stimulus. Then, Western blot was used to evaluate the following proteins: pSTAT3 ^Tyr705^ (**A**), acSTAT3^Lys685^ (**B**), Sirtuin 1 (**C**), pAMPK^Thr172^ (**D**), pCREB^Ser133^ (**E**), pFoxO1^Thr24^/3^Thr32^ (**F**), and POMC (**G**). Protein content was normalized using an antibody against vinculin. Data are expressed as mean ± S.E.M. Two-way ANOVA followed by Bonferroni’s post hoc was performed for group comparison. Filled and empty circles and triangles represent individual plots.

For leptin-induced anorexigenic signaling, α7nAChR^flox^ and α7nAChR^flox^POMC^cre^ mice were fasted overnight, then received an acute injection of saline (0.9%) or leptin (2.5 μg/g BW), and were euthanized 30 min afterward. We then evaluated hypothalamic markers to assess the leptin-mediated anorexigenic response. Intraperitoneal injection of leptin increased hypothalamic pSTAT3^Tyr705^ content in both groups (Figure 11A; drug: *p* < 0.001, genotype: *p* = 0.1730, and interaction: *p* = 0.2733). However, α7nAChR^flox^POMC^cre^ mice showed lower hypothalamic protein levels of acSTAT3^Lys685^ and POMC than those in α7nAChR^flox^ mice (Figure 11F,G; drug: *p* = 0.2861, genotype: *p* = 0.0001, and interaction: *p* = 0.2298). Notably, a decreasing trend in pCREB^Ser133^ content was observed only in leptin-stimulated knockout mice (Figure 11E; drug: *p* = 0.0714, genotype: *p* = 0.054, and interaction: *p* = 0.0417). In addition, intraperitoneal PNU-282987 treatment increased acSTAT3^Lys685^ protein content only in the control group (Supplementary Figure S4B; drug: *p* = 0.0077, genotype: *p* < 0.0001, and interaction: *p* < 0.001). Knockout mice injected with PNU-282987 showed lower pCREB^Ser133^ levels (Supplementary Figure S4C,D; drug: *p* = 0.0142, genotype: *p* = 0.5553, and interaction: *p* = 0.0772). These data suggest that α7nAChR in POMC neurons may contribute to the leptin-induced anorectic response via pCREB^Ser133^. Nevertheless, employing the entire hypothalamus in this experiment presents a limitation.

### 2.4. In Vitro Activation of α7nAChR Induces CREB Phosphorylation and Upregulates Pcsk1 Gene Expression, Suggesting That α7nAChR Functions as a Calcium-Selective Ion Channel in POMC Neurons

**Figure 12:**
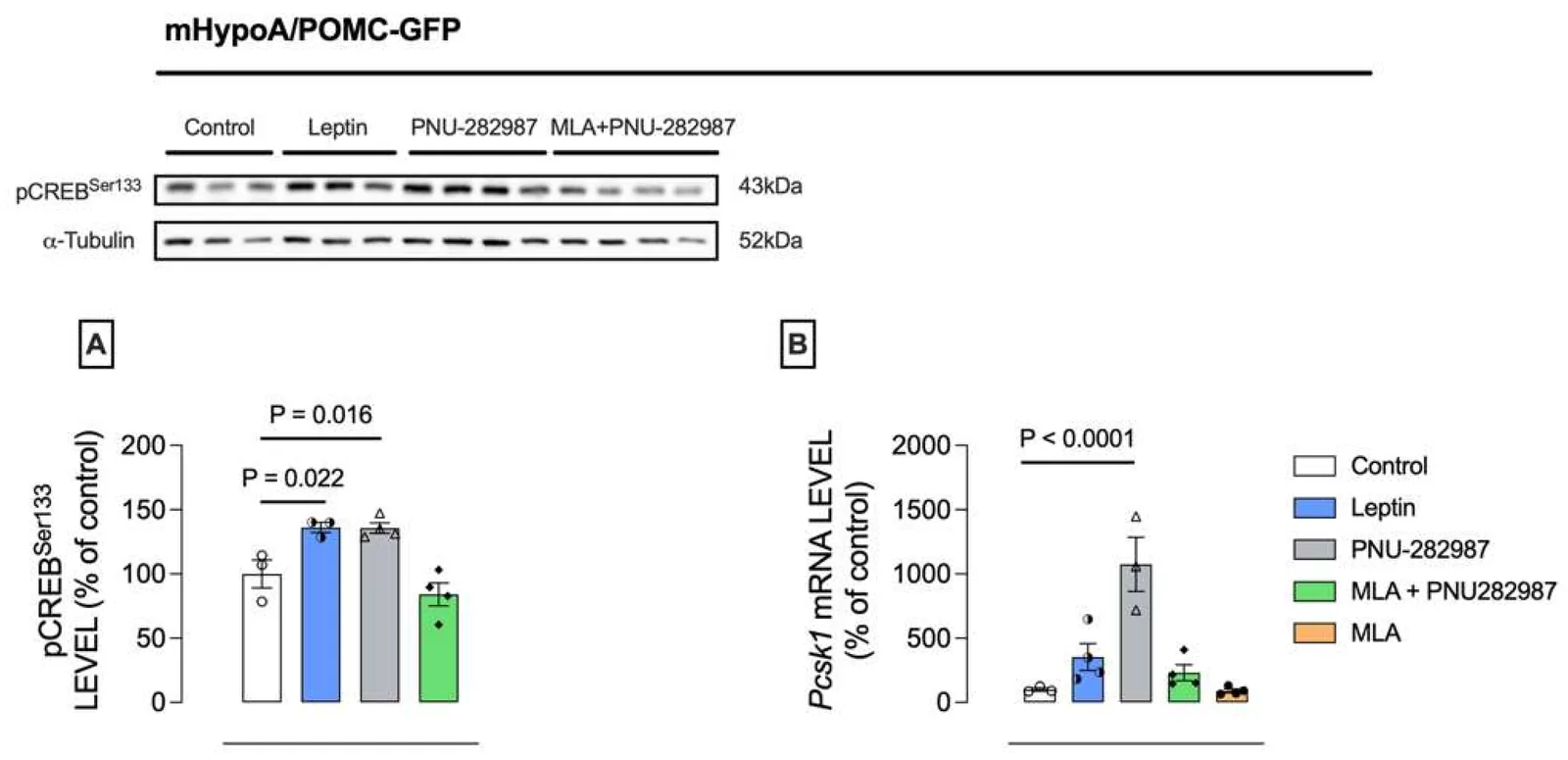
pCREB^Ser133^ protein content and pro-hormone enzyme mRNA content in mHypoA/POMC-GFP cells stimulated by leptin, PNU-282987, and/or MLA. A neuronal cell line from mice (mHypoA/POMC-GFP) was maintained in starvation for 4 h using a medium without FBS. Then, cells were treated with a vehicle (0.9% saline, filtered), leptin (100 ng/mL), PNU-282987 (1 μM), or PNU + MLA (1 μM) for 15 min (*n* = 3–4). Then, we used Western blotting to assess pCREB^Ser133^ levels (**A**) and RT-qPCR to measure *Psck1* expression (**B**). Protein content was normalized using an antibody against α-tubulin, while mRNA expression was normalized by *Actb*. Data are expressed as mean ± S.E.M. One-way ANOVA was performed for comparison among the groups. Each data point represents an independent biological replicate. Filled and empty circles and triangles represent individual plots.

To investigate our hypothesis that the reduction in pCREB^Ser133^, a downstream signaling molecule of calcium activity in POMC neurons, was mediated by impairment of α7nAChR ion channel function, we conducted cell culture experiments. pCREB^Ser133^ levels in mHypoA POMC/GFP cells stimulated with leptin (100 ng/mL) or PNU-282987 (1 μM) were higher than in the control group (Figure 12A). However, treatment with the selective α7nAChR antagonist methyllycaconitine citrate (MLA) inhibited the PNU-282987-induced increase in pCREB^Ser133^ (Figure 12A). In addition, PNU-282987 treatment increased *Pcsk1* mRNA levels, a prohormone enzyme targeted by pCREB^Ser133^ (Figure 12B). However, the selective antagonist reversed this effect (Figure 12B). These findings suggest that pCREB^Ser133^, a transcription factor sensitive to intracellular calcium levels, can be modulated by α7nAChR through its role as a calcium-selective ion channel.

## 3. Discussion

Our primary findings demonstrate that the deletion of α7nAChR in hypothalamic POMC neurons impairs food intake and the leptin anorexigenic response but not glucose homeostasis in young male mice. α7nAChR^flox^POMC^Cre^ mice exhibited increased body weight gain, epididymal fat pad mass, serum leptin levels, and pHSL^Ser565^ at an early age, but no major impairments in glucose homeostasis or insulin signaling were observed. Additionally, these mice showed a trend toward increased food intake during the active cycle and reduced expression of hypothalamic anorexigenic neuropeptide genes. Furthermore, deleting α7nAChR from POMC neurons delayed the leptin-induced anorexigenic response, whereas the effect of PNU-282987 was abolished. This delay may reflect α7nAChR’s role as a calcium-selective ion channel, which reduces pCREB^Ser133^ levels and those of its downstream targets.

Multiple studies indicate that exposure to a high-fat diet (HFD) causes sex-specific metabolic impairments influenced by hormonal regulation and endocrine signaling [29,31,32]. For example, research shows that global α7nAChR deletion does not alter body weight gain (BWG) or adiposity in mice on a control diet in either sex, but it does in those on an HFD [29]. Notably, ovariectomized females fed an HFD exhibited increased BWG and visceral fat, suggesting that ovarian hormones exert protective effects [29]. The link between α7nAChR’s metabolic role and ovarian hormones remains uncertain. Li et al. (2018) found no differences in food intake or body weight in full-body α7nAChR^−/−^ male mice on chow, but these mice gained more weight and showed lower energy expenditure and oxygen consumption on a 45% kcal/lipid HFD for 16 weeks [33]. Other studies show α7nAChR^−/−^ mice have higher body weight, fat accumulation, and food intake even on a chow diet [28]. In addition to its function as a receptor, α7nAChR is a calcium-selective ion channel, and calcium is vital for activating phosphatases that regulate various biological functions, including neuromodulation via hormones such as leptin [34]. Calcium imbalance caused by Opa1 deletion in POMC neurons led to elevated serum leptin, increased food intake, fat deposition, and weight gain [35]. Overall, these findings indicate that deleting α7nAChR in POMC neurons can disrupt energy balance in mice on a normal diet.

Under physiological conditions, acetylcholine is released in a circadian pattern in the hypothalamus, peaking during feeding periods and declining to basal levels afterward [36]. This signaling pathway may support satiety regulation by promoting transcription of anorexigenic neuropeptide genes and inhibiting orexigenic gene expression via the JAK2-STAT3 pathway [25,37]. Therefore, the absence of acetylcholine-activated receptors, such as nicotinic or muscarinic receptors, may disrupt the balance between food intake and energy expenditure [36,38]. As previously shown, HFD consumption reduced hypothalamic α7nAChR expression in mice [23]. Additionally, endogenous molecules, such as leptin, insulin, catecholamines, and acetylcholine, induce calcium influx into POMC neurons, which is essential for neurotransmitter release and for regulating neuronal excitability during synaptic transmission [39,40]. Furthermore, an imbalance in calcium flux can impair the anorexigenic hypothalamic response mediated by mitochondrial reactive oxygen species [35,41]. In this sense, HFD-induced obese mice exhibited reduced neural excitability mediated by calcium flux in POMC neurons [41]. Thus, these impairments may disrupt the regulatory balance between anorexigenic and orexigenic neurons and their projections to brain regions involved in controlling feeding behavior [4].

α7nAChR^−/−^ mice fed a chow diet showed increased area under the curve in ipGTT and ipITT and reduced glucose uptake in the extensor digitorum longus muscle [28]. In contrast, another study found no differences in ipGTT, ipITT, or serum insulin levels in α7nAChR^−/−^ mice fed a chow diet [33]. However, when these mice were exposed to an HFD, the liver showed reductions in pIRS^Tyr162^ and pAKT^Ser473^; insulin resistance was also evident by ipGTT and ipITT [33]. The role of α7nAChR in POMC neurons in hepatic glucose homeostasis remains unknown, and most of that knowledge relies on α7nAChR^−/−^ full-body. POMC neurons also express insulin receptors, enabling them to sense and regulate peripheral glucose levels via vagal efferent signals to peripheral organs (e.g., liver, kidney, and pancreas) [42]. This mechanism is also mediated by α-MSH release, which binds to MC3/4 receptors and regulates efferent and afferent vagal tone to peripheral organs involved in bioprocesses such as gluconeogenesis and insulin secretion [43,44]. As a result, some studies have highlighted the importance of the cholinergic system in central insulin-mediated glucose homeostasis [43,45]. For instance, pharmacological inhibition or deletion of α7nAChR impairs the regulation of gluconeogenic genes in hepatic Kupffer cells and serum glucose levels [45]. Furthermore, deleting MC4R receptors in extrahypothalamic cholinergic neurons (preganglionic neurons) leads to metabolic disruptions, including increases in serum glucose and insulin levels, the glucose infusion rate during an euglycemic clamp, and hepatic glucose production [43]. In our study, deleting α7nAChR in POMC neurons reduces POMC content (the α-MSH precursor) early in life. Furthermore, the reduction in pCREB^Ser133^ levels, a transcription factor that targets genes involved in α-MSH biosynthesis, in response to peripheral hormones could contribute to this impairment in glucose homeostasis. Despite this, we found no major change in glucose homeostasis in the present experimental model.

Leptin acts as a peripheral hormone with a central role in the hypothalamus, signaling the body’s energy stores by crossing the blood–brain barrier and binding to specific populations of neurons (e.g., POMC and NPY neurons). The JAK2-STAT3 pathway is activated when leptin binds to the ObR on POMC neurons, thereby regulating energy balance by increasing the expression of the Pomc and Cartpt genes [46]. The STAT3 transcriptional role appears to depend on its acetylation. The nuclear receptor Nur77 (NR4A1) is involved in STAT3 acetylation by recruiting the p300 enzyme. Inhibition or deletion of Nur77 impairs p300 recruitment, reducing leptin-induced STAT3 acetylation and the anorexigenic response [47]. Our findings suggest that α7nAChR expression in POMC neurons may facilitate leptin-induced anorexigenic responses by modulating STAT3 acetylation. α7nAChR^flox^POMC^Cre^ mice showed reduced hypothalamic STAT3 acetylation levels regardless of leptin stimulation. This finding may suggest that leptin’s ability to trigger STAT3-dependent anorexigenic mechanisms is impaired [47]. The same study observed no significant difference in Pomc gene expression in Nur77 knockout mice following leptin treatment [35,47]. On the other hand, elevated activity of deacetylases such as Sirt1 and HDAC1 can suppress STAT3 transcriptional activity while promoting the nuclear translocation of FOXO1/3a, which bind to the promoters of orexigenic genes (e.g., NPY and AgRP) [37,47,48]. However, Sirt1 protein levels were unchanged between α7nAChR^flox^POMC^Cre^ and α7nAChR^flox^ mice.

Calcium influx through ion channels, which enhances the activity of calmodulin kinase II and IV (CaMKII and IV), is crucial for the leptin-induced anorexigenic response [49]. These enzymes phosphorylate serine and threonine residues on target proteins, including cAMP response element-binding protein (CREB) at serine 133 and CREB-regulated transcription coactivator 1 (*Crtc*) [50,51,52]. Our findings indicate that calcium-selective α7nAChR activity contributes to this process, as leptin and PNU increased pCREB^Ser133^ levels, and MLA (an α7nAChR antagonist) nullified PNU’s effect in vitro. Additionally, PNU elevated prohormone convertase (*Pcsk1*) mRNA expression. Crtc1 and CREB are key transcription factors that translocate to the nucleus to bind the *Cartpt* and prohormone convertase promoters, respectively [15,53], facilitating the conversion of POMC into bioactive molecules [15,53]. In POMC neurons, prohormone convertases 1/3 and 2 (PC1/3 and PC2), along with carboxypeptidase, process pro-opiomelanocortin into α-MSH, β-endorphin, and other smaller molecules [54]. Subsequently, α-MSH binds to melanocortin receptor 4, promoting a negative energy balance by reducing food intake [54,55].

It is important to emphasize that our analyses were conducted across the entire hypothalamus. Therefore, modifications of proteins such as p-CREB Ser^133^, acetyl-STAT3, and p-STAT3, as well as modulation of gene expression and eating behavior, may be influenced by other cell types present in the hypothalamus, such as microglia and astrocytes, or by other neuronal populations that may respond to the stimulus or to deletion of the α7nAChR receptor in POMC neurons. However, our results show that the α7nAChR receptor in POMC neurons plays an important role in the molecular and physiological mechanisms investigated.

## 4. Materials and Methods

### 4.1. Animals and Diet

Mice were housed at 23 ± 1 °C with 50% humidity under a 12 h/12 h light/dark cycle (lights on at 06:00 a.m.) and had ad libitum access to water and a chow diet (Nuvilab^®^, CR-1, Nuvital, Colombo, Paraná, Brazil) [56]. Each mouse was randomly assigned to a cage, and cage positions were randomized as well. To evaluate the role of α7nAChR expressed in hypothalamic POMC neurons in energy balance and the leptin response, mice underwent cell-specific deletion via genetic recombination. These animals were generated by mating Chrna7 floxed mice (B6(Cg)-Chrna7 tm1.1Ehs/YakelJ; Stock n. #005965) with mice expressing the Cre enzyme under the control of the Pomc promoter (Tg (Pomc1-cre) 16Lowl/J; Stock n. #026965). Mice had a C57BL/6J background. We confirmed genotypes by PCR in all animals using the Extract-N-Amp™ tissue PCR Kit (XNAT2; Sigma-Aldrich, St. Louis, MO, USA), and we designed the primers according to Jackson Laboratory protocols. The control group was designated as α7nAChR ^fl/fl^ (*n* = 30; 2–3 animals/cage), while animals with a deletion were designated as α7nAChR^fl/fl^POMC^cre^ (*n* = 32; 2–3 animals/cage). We based the sample size on prior publications. Mouse samples containing less than 1 µg/µL of protein were excluded from the Western blotting analysis, since low protein content may lead to weak or undetectable bands. We measured body weight (BW) weekly and body weight gain (BWG) at the end of the protocol. The present study included males (*n* = 4–6 per group) and females (*n* = 3–5 per group). Due to technical limitations, we did not assess the estrous cycle of female mice during indirect calorimetry or glucose and insulin tolerance tests. Nevertheless, all females were euthanized during estrus. All experiments were conducted in accordance with the ethical guidelines established by the National Council for the Control of Animal Experimentation (CONCEA) and were approved by the Institutional Animal Care and Use Committee (CEUA protocols #5553-1/2020, 5553-1(A)/2021, and 6214-1/2023).

### 4.2. Immunofluorescence

Deletion of α7nAChR in hypothalamic POMC neurons was confirmed by immunofluorescence. Mice were perfused with phosphate-buffered saline (0.9%) and subsequently with paraformaldehyde (4%). The brain was removed and cryosectioned at 15 μm thickness at −30 °C using a cryostat (Leica Biosystems, CM1860, Nussloch, Germany). Slices were washed with Triton X-100 (0.25%), then incubated with hydrogen peroxide (0.3%) for 5 min in a dark chamber. Samples were incubated with albumin (3%) for 2 h. Slices were rewashed and incubated with the α-MSH primary antibody (bs1848R, 1:200; Bioss Antibodies, Woburn, MA, USA). On the second day, slices were incubated with secondary goat anti-rabbit IgG Alexa Fluor 594 (A-11005, 1:500; Thermo Fisher Scientific, Waltham, MA, USA) and α-bungarotoxin conjugated with Alexa Fluor 488 (B13422, 1:500; Thermo Fisher Scientific, MA, USA) for 2 h. The nucleus was stained with DAPI (D1306, 1:500; Thermo Fisher Scientific, MA, USA), and the cover glass was mounted with ProLong^TM^ Gold Antifade Mountant (P36930; Thermo Fisher Scientific, MA, USA). Images were obtained using confocal microscopy at the National Institute of Science and Technology on Photonics Applied to Cell Biology (INFABIC) at the State University of Campinas, USA, on a Zeiss LSM 780-NLO (Carl Zeiss AG, Jena, Germany).

### 4.3. Indirect Calorimetry

Metabolic parameters (e.g., oxygen consumption, carbon dioxide production, respiratory exchange ratio, heat, food intake, and locomotor activity) were measured by indirect calorimetry using the Oxymax-CLAMS (Columbus Instruments, Columbus, OH, USA). At 4 and 8 weeks of life, we individually housed the animals in cages for 24 h for an adaptation period. Afterward, metabolic parameters were measured for 24 h at room temperature (22 ± 1 °C) under a 12 h/12 h light/dark cycle. We analyzed data using CLAX (Columbus Instruments, Columbus, OH, USA).

### 4.4. Glucose Tolerance Test, Insulin Tolerance Test, and Fasting Glucose

We evaluated glucose homeostasis using the glucose tolerance test (GTT), the insulin tolerance test (ITT), and fasting glucose levels. For GTT and ITT, mice were fasted overnight (12 h), fed again (2 h), and then fasted again (4 h) before testing [57]. The GTT measured blood glucose levels at 0 min after an intraperitoneal glucose injection (1 g/kg of BW; G8270, Merck Millipore, Billerica, MA, USA) and at 15, 30, 60, 90, and 120 min using tail blood samples. We calculated the area under the curve (AUC) [57]. The ITT measured blood glucose levels at 0 min, after which we injected insulin (1.5 U/kg BW; Humulin^®^, Eli Lilly and Company, Indianapolis, IN, USA) intraperitoneally. We collected tail blood samples at 5, 10, 15, and 30 min. We calculated the decay constant (kITT) between 10 and 30 min, when glucose levels were consistently decreasing in all animals [57]. Fasting glucose was measured after an overnight fast (12 h) from a tail blood sample. All glucose measurements were performed using an Accu-chek^®^ glucometer, model Active (Roche Diagnostics, Mannheim, Germany). ipGTT and ipITT were performed in the same animals at the same time point, with a 3-day recovery interval between tests.

### 4.5. Food Intake Measurement

Relative food intake was measured using a semi-analytical balance (Marte, AD200 model, São Paulo, Brazil). The animals were individually housed in cages and fasted for 24 h. Body weight was measured to calculate relative food intake, and then acute drug administration was performed. Mice received an intraperitoneal injection of saline 0.9%, leptin (2.5 μg/g body weight), or PNU-282987 (1 mg/kg body weight). Based on leptin clearance and a prior study, we assessed food intake at 1, 2, 3, and 4 h [58,59]. Food intake was defined as the difference between the food weight before and after the offer at each of the described time points. The relative food intake was calculated using the following equation:

$$\mathrm{Relative}\;\mathrm{Food}\;\mathrm{Intake}=\frac{\mathrm{Food}\;\mathrm{intake}\;(g)}{\mathrm{Body}\;\mathrm{weight}\;(g)}$$

### 4.6. Cell Culture Analysis

Murine hypothalamic neurons, mHypoA POMC/GFP (RRID: CVCL_EP69), were immortalized from C57/BL6 mice and kindly provided by Dr. Denise Belsham at the University of Toronto. The cell line was cultured in Dulbecco’s Modified Eagle’s Medium (DMEM; Invitrogen, Carlsbad, CA, USA), low glucose, supplemented with 5% fetal bovine serum (Invitrogen, CA, USA) and 1% penicillin (100 U/mL)/streptomycin (100 μg/mL) (Invitrogen, USA) at 37 °C, 5% CO_2_, and 95% humidity. The cells were starved for 4 h, then treated for 15 min with 100 ng/mL leptin (L3772, Sigma-Aldrich, MO, USA) or 1 μM PNU-282987 (P6499; Sigma-Aldrich, MO, USA). The PNU treatment was performed with or without 1 μM methyllycaconitine citrate (MLA; ab120072). PNU-282987 and MLA were administered simultaneously. Protein was then extracted for Western blotting or RT-qPCR. Each data point is an independent biological replicate.

### 4.7. Drug Treatments

Animals were randomly assigned to acute drug treatment groups (single injection) at the 8th week of life. Different doses were administered to measure food intake. Before euthanasia, animals were fasted for 12 h, and then saline 0.9%, leptin (5 μg/g body weight; Sigma-Aldrich, MO, USA), or PNU-282987 (1 mg/kg body weight; P6499, Sigma-Aldrich, MO, USA) was administered intraperitoneally. Euthanasia was performed 30 min after leptin administration or 45 min after PNU-282987 administration. The summary of the drug treatments is described below (Table 1). All drugs were solubilized in sterile 0.9% saline.

### 4.8. Tissue Collection

At the 4th and 8th weeks of life, the animals were fasted for 12 h. The mice were anesthetized intraperitoneally with ketamine (300 mg/kg of BW) and xilazine (30 mg/kg of BW) and then euthanized by decapitation. Trunk blood was collected and maintained at room temperature (25 °C) for 40 min. Serum was obtained by centrifuging blood (3500 rpm for 15 min). We also collected the whole hypothalamus, epididymal white adipose tissue, brown adipose tissue, and liver and stored them at −80 °C for molecular analyses, including RT-qPCR and Western blotting, as described below. 

### 4.9. Biochemical Analysis

Serum insulin (EMINS, Thermo Fisher Scientific, MA, USA) and leptin levels (MOB00B, R&D Systems, Ann Arbor, MI, USA) were measured by ELISA at the 4th and 8th weeks of life. Insulin ELISA assay sensitivity was 5 μIU/mL, and intra- and inter-assay precision were 3.6% and 7.8%, respectively. Leptin ELISA assay sensitivity was 5.5 pg/mL, and intra- and inter-assay precision were <10% and <12%, respectively. We performed the assays according to the manufacturer’s instructions.

### 4.10. Real-Time Quantitative PCR

RNA was extracted using TRIzol reagent (Invitrogen Corporation, CA, USA) according to the manufacturer’s instructions and quantified using a NanoDrop ND2000 spectrophotometer (Thermo Electron, Madison, WI, USA). We assessed integrity by agarose gel electrophoresis (1.5%) and the absence of genomic DNA. Reverse transcription was performed with 3 µg of total RNA using a High-Capacity cDNA Reverse Transcription kit (4368813; Life Technologies Corporation, Carlsbad, CA, USA). To evaluate mRNA expression levels, fluorogenic probes were used, including Pomc (Mm00435874_m1), *Npy* (Mm01410146_m1), *Cartpt* (Mm04210469_m1), *Agrp* (Mm00475829_g1), *Prkaa2* (Mm01264789_m1), *Pcsk1* (Mm01345253_m1), and *Actb* (4351315). Reactions were performed in triplicate. Real-time PCR was performed on an AB/Prism 7500 fast platform. Data were analyzed using the Sequence Detection System 2.0.5.

### 4.11. Western Blotting

The whole hypothalamus, white and brown adipose tissues, and the liver were homogenized in ice-cold buffer containing 1% *v*/*v* Triton X-100, 0.1 mol/L Tris (pH 7.4), 0.1 mol/L sodium pyrophosphate, 0.1 mol/L sodium fluoride, 0.01 mol/L EDTA, 0.01 mol/L sodium vanadate, 0.002 mol/L PMSF, and 0.01 mg/mL aprotinin. The supernatant protein was obtained by centrifugation (10,500 rpm/30 min) at 4 °C, and the protein content was determined. Samples were excluded when they could not be quantified, or when protein content was <1 µg/µL. The samples were resuspended in the Laemmli sample buffer. Protein separation was performed on SDS-PAGE using a small gel apparatus (BioRad, Richmond, CA, USA). We transferred proteins for 2 h at 120 volts using a transfer buffer containing methanol and SDS. Nitrocellulose membranes were incubated overnight at 4 °C with specific antibodies such as pIRS^Ser307^ (#2381; 1:1000), pSTAT3^Tyr705^ (#9145S; 1:1000), UCP1 (#14670; 1:1000), pAMPK^Thr172^ (#2535S; 1:1000), pFOXO1^Thr24^/3^Thr32^ (#9464; 1:1000), pHSL^Ser565^ (#4137; 1:1000), pAKT^Ser473^ (#4060; 1:2000), pCREB^Ser133^ (#9198; 1:1000), PI3K (#4292; 1:1000), PPAR- γ (#2443; 1:1000), Sirtuin 1 (#2310S; 1:1000), and vinculin (#139015; 1:2000) (Cell Signaling, Danvers, MA, USA), CPT2 (sc-377294; 1:1000), G6Pase (sc-25840; 1:1000), PEPCK (sc166778; 1:1000), GAPDH (sc-47724; 1:1000) (Santa Cruz Technology, Dallas, TX, USA), α-Tubulin (T5168; 1:5000) (Sigma-Aldrich, MI, USA), and Acetyl-STAT3^Lys685^ (PA5-17429; 1:1000) (Thermo Fisher, MA, USA). Then, after a Tris-buffered saline (TBS)-Tween 20 (TTBS; 10 mmol/L Tris, 150 mmol/L NaCl, 0.5% Tween 20) wash, the nitrocellulose membranes were probed with goat peroxidase-conjugated secondary antibodies (KPL, Gaithersburg, MD, USA) for 2 h at room temperature. Proteins were detected by a chemiluminescence kit (SuperSignal West Pico Chemiluminescent Substrate, Thermo Fisher Scientific, MA, USA), and bands were evaluated by densitometry using Scion Image 4.0 software (ScionCorp, Frederick, MD, USA). The housekeeping genes used to validate the amount of protein loaded on the gel were selected based on the tissue. The housekeeping proteins used are described in the figure legend. Undetectable bands were not quantified.

### 4.12. Statistical Analysis

We analyzed the data using GraphPad Prism 7.0 software (La Jolla, USA). We assessed the normality of the data using the Shapiro–Wilk test. All data were parametric and evaluated using Welch’s *t*-test or Two-way ANOVA (time × genotype or drug treatment × genotype), followed by Bonferroni’s post hoc test when necessary. Data were expressed as mean ± standard error of the mean. The level of significance was maintained at 5% (*p* < 0.05).

## 5. Conclusions

In summary, α7nAChR expression in hypothalamic POMC neurons influences energy balance. Its absence leads to early weight gain and increased fat in male mice, along with decreased levels of anorexigenic neuropeptides in the hypothalamus. Furthermore, central α7nAChR appears to help regulate liver glucose levels and insulin sensitivity. Additionally, α7nAChR is crucial for leptin-driven appetite suppression, possibly due to a trend toward reduced levels of pCREBSer133, a key transcription factor for enzymes involved in central anorexigenic pathways. These results suggest that α7nAChR functions as a calcium-permeable ion channel.

## 6. Limitations and Future Steps

The present study has limitations that future studies can address. The mouse line Tg(Pomc1-cre)16Lowl used in this study has limitations, which are stated below. A subset of POMC neurons differentiates into AgRP/NPY neurons during development in different brain regions, including the hypothalamus, which might affect the results. Furthermore, Pomc-Cre mice may exhibit some physiological alterations that were not evaluated here, since only Flox/Flox animals were used as controls. Our study shows that females had different outcomes than males, suggesting a possible hormonal effect. Future studies using females with α7nAChR deletion in POMC neurons and subjected to ovariectomy would provide further insight into how hormones interact with α7nAChR in energy balance and glucose homeostasis. Furthermore, to determine whether the calcium-selective ion channel is relevant to the leptin anorexigenic response in the hypothalamus, cell-targeted electrophysiological recordings from these POMC neurons in the α7nAChR^flox^POMC^Cre^ mouse line would be necessary, as would calcium imaging in the presence of leptin and/or an α7nAChR-selective antagonist.

## Figures and Tables

**Table 1:** Summary of drug treatments used in the present study.

Drug	Dose/Concentration	Experiment	Model	Reference *
Leptin	5 μg/g of BW	Food intake	In vivo	[60]
	2.5 μg/g of BW	Western blotting	In vivo	[58]
	100 ng/mL	Western blotting/PCR	In vitro	[61]
PNU-282987	1 mg/kg of BW	Western blotting	In vivo	[62]
	1 mg/kg of BW	Food intake	In vivo	[62]
MLA	1 uM	Western blotting/PCR	In vitro	[63]

* Adapted from the paper.

## Data Availability

The original contributions presented in this study are included in the article/Supplementary Materials. Further inquiries can be directed to the corresponding author.

## Supplementary Materials

The following supporting information can be downloaded at: https://www.mdpi.com/article/10.3390/ijms27198697/s1.
