# Supplementary material for: Deletion of α7 Nicotinic Acetylcholine Receptor in POMC Neurons Impairs Energy Balance and Leptin-Induced Anorexigenic Response in Male Mice
Source: Int J Mol Sci. 2026 Sep 29;27(19):8697. doi: 10.3390/ijms27198697 (PMC13664977; doi:10.3390/ijms27198697)
Supplement: Supplementary file 1 [file ijms-27-08697-s001.zip › ijms-4495711-supplementary.pdf]

Supplementary data

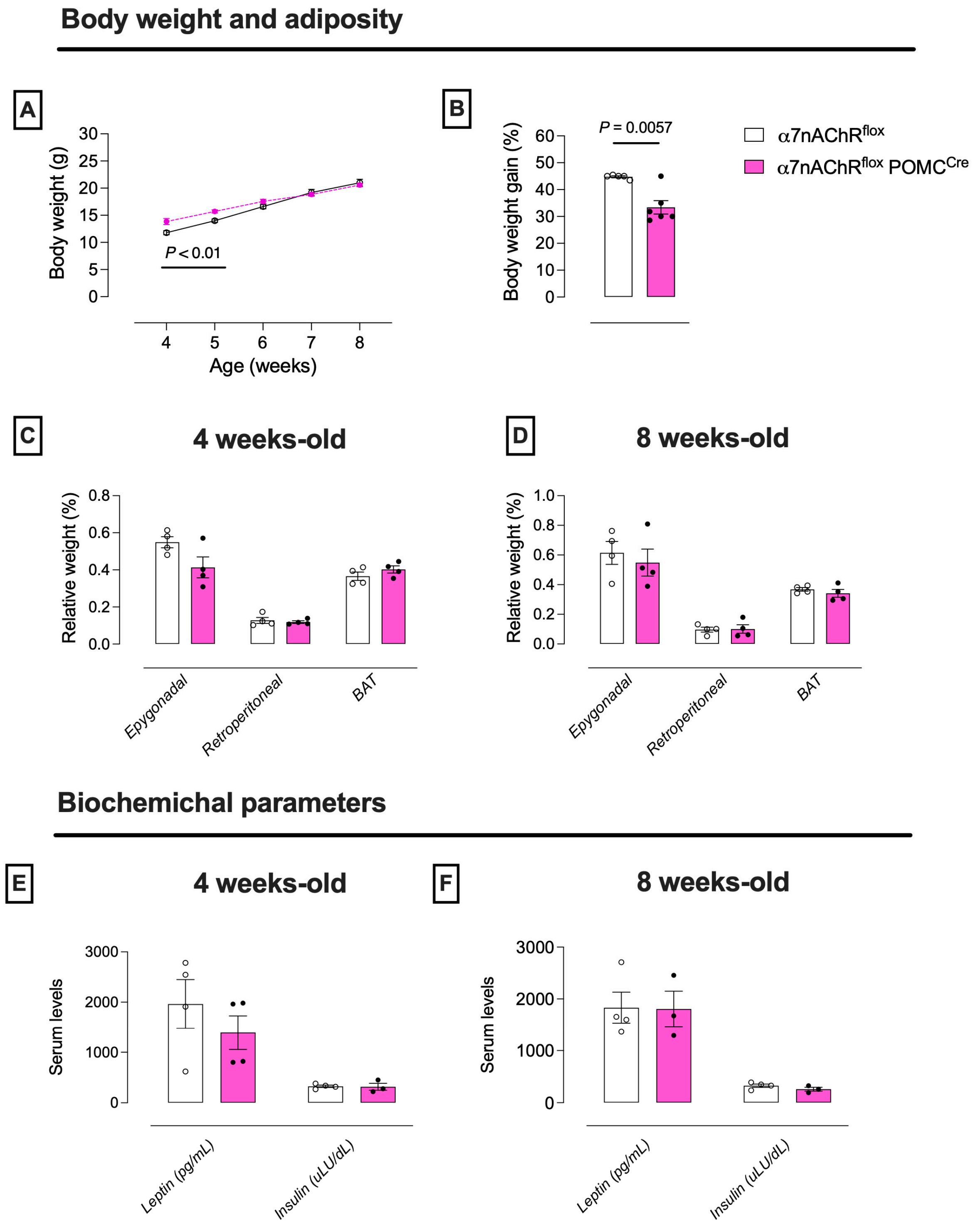

Figure S1. Growth parameters, adiposity, leptin, and insulin serum levels in females at the 4th and 8th week of life. Body weight (A) was measured weekly from the 4th until the 8th week of life, while body weight gain (B) was measured at the 8th week of life (n=5-6). Adiposity was determined by weighing the epigonadal, retroperitoneal, and brown adipose tissue (C-D) (n=3-4). Leptin and insulin serum levels were determined using ELISA kits at the same ages (E-F) (n=3-4). Data were expressed as mean ± S.E.M., Welch's t-test was performed.

Metabolic parameters 4 week-old

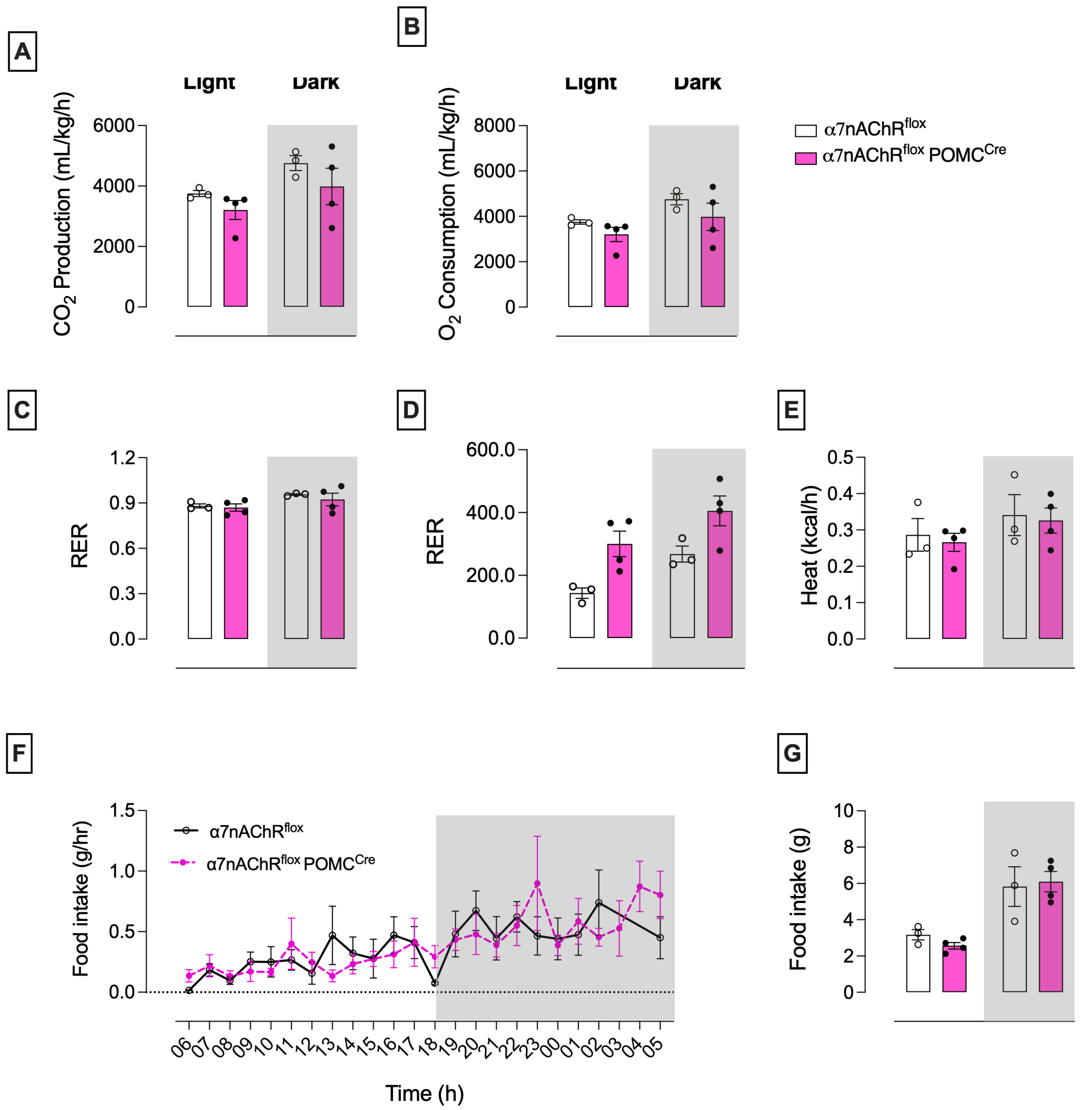

Figure S2. Metabolic parameters *in females* at the 4th week of life. Metabolic parameters, including oxygen consumption (A), carbon dioxide production (B), RER (C), locomotor activity (D), heat (E), and relative food intake (F-G), were measured by indirect calorimetry for 24 hours (n=3-4). Data were expressed as mean ± S.E.M.; Welch's t-test was performed.

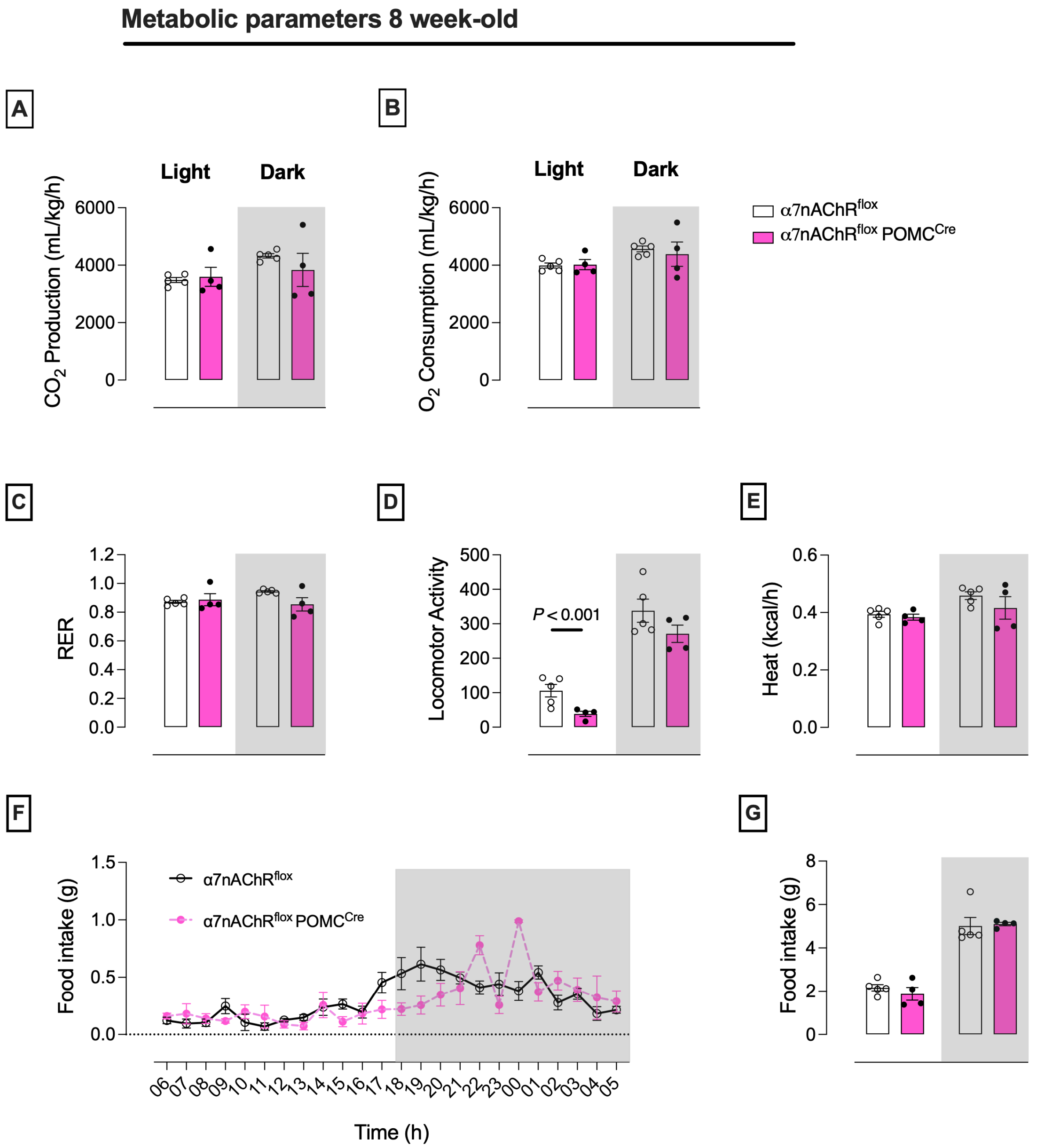

Figure S3. Metabolic parameters were measured at the 8th week of life in females. Metabolic parameters, including oxygen consumption (A), carbon dioxide production (B), RER (C), locomotor activity (D), heat (E), and relative food intake (F-G), were measured by indirect calorimetry for 24 hours (n=4-5). Data were expressed as mean ± S.E.M., Welch's t-test was performed.

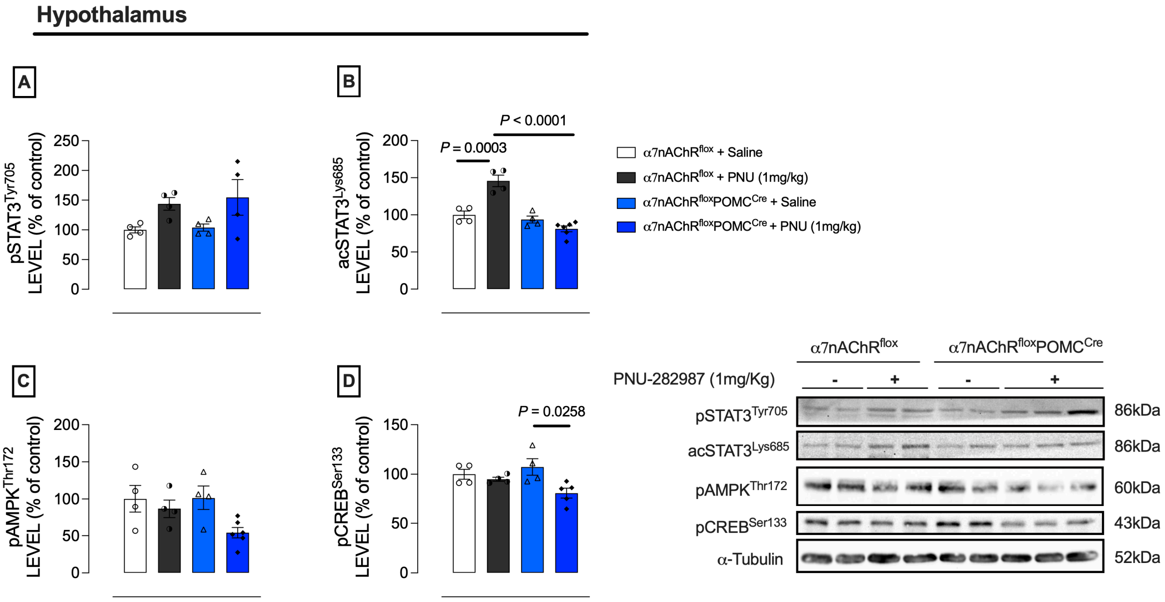

Figure S4. Hypothalamic protein involved in the anorexigenic response to PNU-282987 administered by intraperitoneal injection. Mice were fasted overnight and then received a single dose of saline solution (0.9%) or PNU-282987 (0.6 mg/Kg BW) by intraperitoneal injection (n=4-6). The hypothalamus was collected 45 minutes after the stimulus. Protein content was normalized using an antibody against α-tubulin. Data are expressed as mean ± S.E.M. Two-way ANOVA was used for group comparison. Samples were excluded when they could not be quantified.
